# Effect of Water, Sanitation, Handwashing, and Nutrition Interventions on Enteropathogens in Children 14 Months Old: A Cluster-Randomized Controlled Trial in Rural Bangladesh

**DOI:** 10.1093/infdis/jiaa549

**Published:** 2020-08-29

**Authors:** Jessica A Grembi, Audrie Lin, Md Abdul Karim, Md Ohedul Islam, Rana Miah, Benjamin F Arnold, Elizabeth T Rogawski McQuade, Shahjahan Ali, Md Ziaur Rahman, Zahir Hussain, Abul K Shoab, Syeda L Famida, Md Saheen Hossen, Palash Mutsuddi, Mahbubur Rahman, Leanne Unicomb, Rashidul Haque, Mami Taniuchi, Jie Liu, James A Platts-Mills, Susan P Holmes, Christine P Stewart, Jade Benjamin-Chung, John M Colford, Eric R Houpt, Stephen P Luby

**Affiliations:** Division of Infectious Diseases and Geographic Medicine, Stanford University, Stanford, California, USA; Division of Epidemiology and Biostatistics, School of Public Health, University of California, Berkeley, Berkeley, California, USA; Infectious Diseases Division, International Centre for Diarrhoeal Disease Research, Bangladesh, Dhaka, Bangladesh; Infectious Diseases Division, International Centre for Diarrhoeal Disease Research, Bangladesh, Dhaka, Bangladesh; Infectious Diseases Division, International Centre for Diarrhoeal Disease Research, Bangladesh, Dhaka, Bangladesh; Francis I. Proctor Foundation, University of California, San Francisco, San Francisco, California, USA; Division of Infectious Diseases and International Health, University of Virginia, Charlottesville, Virginia, USA; Infectious Diseases Division, International Centre for Diarrhoeal Disease Research, Bangladesh, Dhaka, Bangladesh; Infectious Diseases Division, International Centre for Diarrhoeal Disease Research, Bangladesh, Dhaka, Bangladesh; Infectious Diseases Division, International Centre for Diarrhoeal Disease Research, Bangladesh, Dhaka, Bangladesh; Infectious Diseases Division, International Centre for Diarrhoeal Disease Research, Bangladesh, Dhaka, Bangladesh; Infectious Diseases Division, International Centre for Diarrhoeal Disease Research, Bangladesh, Dhaka, Bangladesh; Infectious Diseases Division, International Centre for Diarrhoeal Disease Research, Bangladesh, Dhaka, Bangladesh; Infectious Diseases Division, International Centre for Diarrhoeal Disease Research, Bangladesh, Dhaka, Bangladesh; Infectious Diseases Division, International Centre for Diarrhoeal Disease Research, Bangladesh, Dhaka, Bangladesh; Infectious Diseases Division, International Centre for Diarrhoeal Disease Research, Bangladesh, Dhaka, Bangladesh; Infectious Diseases Division, International Centre for Diarrhoeal Disease Research, Bangladesh, Dhaka, Bangladesh; Division of Infectious Diseases and International Health, University of Virginia, Charlottesville, Virginia, USA; Division of Infectious Diseases and International Health, University of Virginia, Charlottesville, Virginia, USA; Division of Infectious Diseases and International Health, University of Virginia, Charlottesville, Virginia, USA; Department of Statistics, Stanford University, Stanford, California, USA; Institute for Global Nutrition, University of California, Davis, Davis, California, USA; Division of Epidemiology and Biostatistics, School of Public Health, University of California, Berkeley, Berkeley, California, USA; Division of Epidemiology and Biostatistics, School of Public Health, University of California, Berkeley, Berkeley, California, USA; Division of Infectious Diseases and International Health, University of Virginia, Charlottesville, Virginia, USA; Division of Infectious Diseases and Geographic Medicine, Stanford University, Stanford, California, USA

**Keywords:** enteric pathogens, water, sanitation, and handwashing, WSH, nutrition, Bangladesh, child health

## Abstract

**Background:**

We evaluated the impact of low-cost water, sanitation, and handwashing (WSH) and child nutrition interventions on enteropathogen carriage in the WASH Benefits cluster-randomized controlled trial in rural Bangladesh.

**Methods:**

We analyzed 1411 routine fecal samples from children 14 ± 2 months old in the WSH (n = 369), nutrition counseling plus lipid-based nutrient supplement (n = 353), nutrition plus WSH (n = 360), and control (n = 329) arms for 34 enteropathogens using quantitative polymerase chain reaction. Outcomes included the number of co-occurring pathogens; cumulative quantity of 4 stunting-associated pathogens; and prevalence and quantity of individual pathogens. Masked analysis was by intention-to-treat.

**Results:**

Three hundred twenty-six (99.1%) control children had 1 or more enteropathogens detected (mean, 3.8 ± 1.8). Children receiving WSH interventions had lower prevalence and quantity of individual viruses than controls (prevalence difference for norovirus: –11% [95% confidence interval {CI}, –5% to –17%]; sapovirus: –9% [95% CI, –3% to –15%]; and adenovirus 40/41: –9% [95% CI, –2% to –15%]). There was no difference in bacteria, parasites, or cumulative quantity of stunting-associated pathogens between controls and any intervention arm.

**Conclusions:**

WSH interventions were associated with fewer enteric viruses in children aged 14 months. Different strategies are needed to reduce enteric bacteria and parasites at this critical young age.

## Teaser

We evaluated the impact of water, sanitation, and handwashing (WSH) and nutrition interventions on enteropathogens in children in Bangladesh. WSH interventions were associated with fewer enteric viruses compared to controls. There was no difference in bacterial and protozoan pathogens for WSH or nutrition interventions.

Diarrheal disease remains the fifth leading cause of child mortality, with the highest burdens in low- and middle-income countries [1]. Children who survive often have persistent deficits in physical growth and cognitive development [2–4]. Asymptomatic polymicrobial pathogen carriage is common in areas with high diarrheal disease burden [5, 6]. In some cases, subclinical pathogen carriage had stronger negative dose-dependent associations with child growth than pathogen-associated diarrhea [5–8]. Mucosal damage induced by specific enteropathogens has also been correlated with decreased performance of oral vaccines, again irrespective of diarrheal symptoms [9, 10].

Interventions targeting household drinking water, sanitation, and handwashing (WSH) practices aim to reduce fecal-oral transmission of enteropathogens in areas without municipal water and sewerage. Household WSH trials have reported varied success in reducing diarrhea and/or specific enteropathogens [11–14]. WSH interventions combined with child-specific nutrition interventions could be synergistic in improving growth, especially when dietary diversity and caloric intake are limited. Nutritional interventions might support improved gut mucosal immune function and a more resilient commensal gut microbiota to provide colonization resistance and pathogen clearance after infection [15, 16]. However, there is potential for nutritional interventions supplying iron to provide a growth advantage to iron-scavenging pathogens over beneficial commensals that promote intestinal barrier function [17]. Meta-analyses of multiple-micronutrient fortification of complementary foods, covering mostly low-income countries across Asia, Africa, and the Americas over the past decade, report no impact or increased risk of diarrhea [18–20]. None of these studies measured enteropathogens.

Direct measurement facilitates objective assessment of pathogen carriage [21]. Most studies have been limited by the measurement of a small number of enteropathogens. Only the Sanitation, Hygiene, Infant Nutrition Efficacy (SHINE) trial in Zimbabwe has evaluated the impact of WSH, child nutrition, and combined child nutrition and WSH (N+WSH) interventions on a comprehensive suite of enteropathogens. Compared with controls, no intervention reduced enteropathogen prevalence or quantity in children 6–12 months old, indicating interventions might have been insufficient to disrupt pathogen transmission [13].

The WASH Benefits Bangladesh cluster-randomized controlled trial reported 31%–38% relative reductions in child diarrhea from WSH, nutrition, and N+WSH interventions; and 0.13–0.25 higher length-for-age *z* scores at 22 months of age for children receiving nutrition and N+WSH interventions compared with controls [22]. A sister trial in Kenya found comparable growth improvements for children receiving nutrition and N+WSH interventions but no reduction in diarrhea for any group, despite similar interventions and sample size, results consistent with the SHINE trial [23]. In Bangladesh, health promoters had more frequent contact with study households and intervention uptake was higher than the other trials [23]. An evaluation of 6 enteric parasites in children 30 ± 2 months old from the Bangladesh trial reported lower prevalence of *Giardia* and hookworm in WSH and N+WSH arms than controls [24, 25]. In Kenya, children from the WSH and N+WSH arms had lower *Ascaris* prevalence than controls [26]. These findings suggest that WSH interventions can reduce parasites in young children; however, the leading etiologies of diarrheal disease for children <5 years old are bacterial and viral [27, 28].

This study assessed the impact of WSH, nutrition, and N+WSH interventions on 34 bacterial, viral, and parasitic enteropathogens in a subset of WASH Benefits Bangladesh children 14 ± 2 months of age. We evaluated the impact of interventions on prevalence and quantity of individual pathogens, as well as composite measures: the number of unique enteropathogens detected, in total and stratified by bacteria, viruses, and parasites; and a novel measure of the quantity of the 4 most prominent stunting-associated pathogens from the Etiology, Risk Factors, and Interactions of Enteric Infections and Malnutrition and the Consequences for Child Health and Development Project (MAL-ED) (ie, enteroaggregative *Escherichia coli* [EAEC], *Shigella*/enteroinvasive *E. coli* [EIEC], *Campylobacter* spp, and *Giardia* spp) [5].

## MATERIALS AND METHODS

### Study Design and Population

The WASH Benefits Bangladesh study measured the impact of WSH and nutrition interventions on child growth, development, and parasitic infection (ClinicalTrials.gov, NCC01590095) [22, 24, 25, 29, 30]. Intervention arms included drinking water treatment, sanitation, handwashing, combined WSH, nutrition, and combined N+WSH. This analysis includes children enrolled in the environmental enteric dysfunction substudy, which was a subsample of clusters from the WSH, nutrition, N+WSH, and control arms (in a 1:1:1:1 ratio) [31, 32]. Interventions were delivered while mothers were pregnant, so children were exposed to interventions from birth; a detailed description of interventions has been provided previously [22]. In brief, the WSH intervention consisted of chlorine tablets and a safe drinking water storage vessel; a dual-pit latrine with a water seal, child potties, and hoes for feces disposal; and handwashing stations (including detergent soap with dispensers) near the latrine and kitchen. The nutrition intervention consisted of age-appropriate infant feeding recommendations plus lipid-based nutrient supplements twice daily from age 6 months to 24 months. The N+WSH intervention combined the WSH and nutrition packages. Behavior change messaging was delivered 6 times per month to intervention households, which resulted in high adherence [22]. Rotavirus vaccination had not been implemented in Bangladesh at the time of the study.

Although enteropathogen prevalence tends to increase with age over the first 2 years of life, the importance of early pathogen carriage on later health outcomes (eg, stunting and cognitive deficits [5, 33]) motivated us to evaluate children at a younger age (14 ± 2 months) than the parasite studies (30 ± 2 months) [31, 32]. Written informed consent was obtained from parents of all children. The trial was approved by human subjects committees at the International Centre for Diarrhoeal Disease Research, Bangladesh (icddr,b; PR-11063), the University of California, Berkeley (2011-09-3652), and Stanford University (25863).

### Fecal Sample Collection, Total Nucleic Acid Extraction, and Pathogen Quantification

Primary caregivers collected child fecal samples, which were placed on cold chain 155 minutes (interquartile range, [IQR], 80–529) after defecation, transported on dry ice to the laboratory, and stored at –80°C. DNA and RNA were extracted using the QIAamp Fast DNA Stool Mini kit (Qiagen, Venlo, The Netherlands) and a modified protocol, which included spike-ins of 2 extrinsic controls to monitor extraction and amplification efficiency [34]. Enteropathogens (Supplementary Table 1) were measured via quantitative polymerase chain reaction (PCR) using a TaqMan array card (validation, conditions, and quality controls measures reported previously [28, 34]) at icddr,b. Quantification cycle value was used as an inverse measure of pathogen quantity, with 1 unit corresponding to a doubling of pathogen quantity and the analytical limit of detection at quantification cycle 35 [27]. Pathogen quantities were normalized based on per-sample extraction/amplification efficiency. For nondetects, quantity was set to half of the detection limit (1.8495 log_10_ copies/g of stool).

### Statistical Analysis

Analysis was by intention-to-treat. Analysis plans describing pathogen outcomes were prespecified (see https://osf.io/ky275/). This study did not target collection of diarrheal stools. The subset of stools from children with reported diarrheal symptoms in the previous 7 days was small and not representative of all diarrhea episodes, which prohibited an analysis of diarrheal stools. Therefore, our analysis focuses on outcomes of enteropathogen carriage in children, including both symptomatic and asymptomatic pathogen detection. We also conducted 3 post hoc analyses that (1) excluded samples from children with reported diarrheal symptoms, (2) evaluated seasonal effect modification of intervention efficacy, and (3) adjusted for only child age and season of sample collection. Investigators were masked to group assignment until primary analysis was complete. Statistical analyses were performed in R software (version 3.5.2) [35].

Composite outcomes included the number of pathogens in total and by type (bacteria, virus, parasite) and an aggregate metric for the quantity of 4 stunting-associated organisms (EAEC, *Shigella*/EIEC, *Campylobacter* spp, *Giardia* spp) that were independently associated with linear growth deficits in a longitudinal analysis of the MAL-ED study where the strength of association was robust to the presence of other enteropathogens [5]. We used g-computation [13] to estimate the absolute difference in the outcome between study arms (using Poisson regression for the number of pathogens and linear regression for the composite stunting-associated pathogen quantity). We obtained confidence intervals (CIs) with a nonparametric bootstrap (B = 1000) that resampled clusters with replacement.

For the 18 pathogens detected in *>*5% of samples, we estimated prevalence differences and ratios between study arms using generalized linear models with robust standard errors [22, 31]. We also estimated differences in log_10_ quantity using the g-computation estimator with a 2-component model, including logistic and log-linear regression steps to account for the sparse, semicontinuous quantity data [5, 13]. In addition to 95% CIs, we report *P* values corrected for multiple comparisons using the Benjamini–Hochberg procedure [36] within each treatment contrast and model type. For quantity analyses, we employed a double bootstrap (B = 1000 for outer, and BB = 25 for inner bootstrap) to estimate *P* values on g-computation results before applying the Benjamini–Hochberg correction.

We prespecified a fully adjusted analysis to improve precision of estimates and control for potential residual confounding from baseline and sample-specific covariates (Supplementary Table 3). Covariates for adjusted analyses were prescreened with a likelihood ratio test, and those with *P* < .1 in bivariate analysis with the outcome were retained for the fully adjusted model. A final analysis accounted for differential missingness of the outcome due to incomplete sample collection using data-adaptive, targeted maximum likelihood estimation, and inverse probability of censoring weighting [37].

## RESULTS

Of 1532 eligible children, 1411 fecal samples with valid qPCR results were obtained from children aged 14 ± 2 months enrolled in the WSH (n = 369), nutrition (n = 353), N+WSH (n = 360), and control (n = 329) arms of the WASH Benefits Bangladesh trial (110 did not provide a fecal sample; 21 did not provide sufficient sample). Diarrheal symptoms were reported in the past 7 days for 214 (14.0%) children, and the proportion of children who did not provide a fecal sample was not different between those with (0.06 [95% CI, .03–.10]) or without (0.07 [95% CI, .06–.09]) reported diarrheal symptoms. Household enrollment characteristics were balanced across study arms and were similar to the main trial (Table 1 and Supplementary Table 2). Fewer children from the control arm provided fecal samples, and there was imbalance across study arms for 2 prognostic covariates related to sample collection: season (monsoon vs dry; Supplementary Figure 1) and child age (Welch’s *t* test between control and each intervention arm: season *P* < .006, age *P < .*0001; Table 1). Several bacterial enteropathogens were more prevalent during the monsoon season across all arms (Supplementary Figure 2). Imbalance by arm and strong relationship between enteropathogen carriage and both monsoon season and age led us to rely on adjusted analyses for primary inference, according to our analysis plan.

**Table 1.** Household Enrollment and Child/Sample Characteristics by Intervention Arm

Characteristic	Control (n = 499)	WSH (n = 446)	Nutrition (n = 435)	N+WSH (n = 447)
Household enrollment characteristics				
Maternal				
Age, y	23 (5)	24 (5)	24 (5)	24 (5)
Height, cm	151 (5)	150 (5)	150 (6)	150 (5)
Years of education	7 (3)	6 (3)	6 (4)	6 (3)
Paternal				
Years of education	5 (4)	5 (4)	5 (4)	5 (4)
Works in agriculture	104 (23%)	128 (29%)	148 (34%)	127 (28%)
Household				
No. of people	5 (2)	5 (2)	5 (2)	5 (2)
No. of children <18 y	2 (1)	2 (1)	2 (1)	2 (1)
Has electricity	269 (60%)	278 (62%)	269 (62%)	272 (61%)
Has a cement floor	75 (17%)	55 (12%)	50 (11%)	53 (12%)
Acres of agricultural land owned	0.18 (0.25)	0.17 (0.26)	0.17 (0.30)	0.13 (0.18)
Drinking water				
Shallow tubewell is primary water source	329 (73%)	337 (76%)	309 (71%)	318 (71%)
Has stored water at home	230 (51%)	199 (45%)	209 (48%)	229 (51%)
Reported treating water yesterday	1 (0%)	0 (0%)	0 (0%)	1 (0%)
Minutes to primary drinking water source	1 (2)	1 (6)	1 (2)	1 (2)
Sanitation				
Daily defecating in the open				
Adult men	19 (4%)	29 (7%)	38 (9%)	38 (9%)
Adult women	12 (3%)	16 (4%)	23 (5%)	21 (5%)
Children aged 8 to <15 y	9 (5%)	17 (8%)	13 (8%)	22 (11%)
Children aged 3 to <8 y	65 (30%)	89 (37%)	90 (40%)	92 (37%)
Children aged 0 to <3 y^a^	71 (72%)	73 (75%)	68 (80%)	79 (88%)
Latrine				
Owned^b^	271 (60%)	244 (55%)	234 (54%)	230 (51%)
Concrete slab	426 (97%)	400 (93%)	382 (93%)	399 (94%)
Functional water seal	157 (38%)	95 (26%)	114 (32%)	111 (31%)
Visible stool on slab or floor	197 (45%)	225 (54%)	210 (52%)	222 (53%)
Owned a child potty	37 (8%)	20 (4%)	27 (6%)	21 (5%)
Human feces observed				
In the house	25 (6%)	36 (8%)	41 (9%)	36 (8%)
In the child’s play area	5 (1%)	4 (1%)	7 (2%)	6 (1%)
Handwashing location				
Within 6 steps of latrine				
Has water	84 (21%)	51 (13%)	38 (10%)	54 (13%)
Has soap	45 (11%)	32 (8%)	23 (6%)	27 (7%)
Within 6 steps of kitchen				
Has water	48 (12%)	40 (10%)	43 (11%)	42 (10%)
Has soap	18 (4%)	11 (3%)	19 (5%)	14 (3%)
Nutrition				
Household is food secure^c^	331 (74%)	298 (67%)	308 (71%)	317 (71%)
Child/sample characteristics				
Female child	225 (50%)	238 (53%)	228 (52%)	214 (47%)
Child stool sample collected	377 (84%)	394 (88%)	379 (87%)	380 (84%)
Child diarrhea reported at stool collection visit	67 (18%)	43 (11%)	61 (16%)	43 (11%)
Child age at stool collection, d	455 (66)	415 (57)	424 (54)	417 (57)
Sample collected during monsoon season^d^	155 (41%)	283 (72%)	244 (64%)	269 (71%)
Time until sample placed on cold chain, min^e^, median (IQR)	160 (76–767)	159 (79–495)	148 (80–398)	158 (90–525)

Values represent either the mean (standard deviation) or No. (%) unless otherwise indicated.

Abbreviations: IQR, interquartile range; N+WSH, nutrition plus water, sanitation, and handwashing; WSH, water, sanitation, and handwashing.

^a^Open defecation does not include diaper disposal of feces.

^b^Households that do not own a latrine typically share a latrine with extended family members who live in the same compound.

^c^Assessed by the Household Food Insecurity Access Scale.

^d^Monsoon season is May–October.

^e^Time between when caregiver reported collecting child’s fecal sample and field staff received sample and placed it on cold chain; values were not normally distributed.

Only 18 organisms were detected in *>*5% of stool samples (Supplementary Figure 3). The most prevalent bacteria were EAEC (76%), *Campylobacter* spp (47%), atypical enteropathogenic *E. coli* (41%), and enterotoxigenic *E. coli* (40%; 20% each for heat-labile and heat-stable toxin, 5% of samples had both). The most prevalent viruses were norovirus (17%; 15% for genogroup GII, 3% for GI, 1% of samples had both), sapovirus (12%), and adenovirus 40/41 (8%). Rotavirus was present in 2.2% of all samples, and 4.0% of samples where caregivers reported diarrhea in the past 7 days. The parasites detected at >5% prevalence were *Giardia* (14%), *Cryptosporidium* (12%), and *Enterocytozoon bieneusi* (10%).

### Impact of Interventions on Composite Pathogen Load

Enteropathogen carriage was high: 326 (99.1%) control children had at least 1 pathogen detected, with an average of 3.8 (standard deviation [SD], 1.8) co-occurring pathogens: 2.8 (SD, 1.4) bacteria, 0.6 (SD, 0.6) viruses, and 0.4 (SD, 0.7) parasites. Children in the WSH group had 0.50 (95% CI, .07–.90) fewer total pathogens and 0.28 (95% CI, .09–.48) fewer total viruses compared with control children (Table 2). Children who received nutritional interventions had fewer total viruses than controls (0.17 [95% CI, .05–.38] for nutrition; 0.21 [95% CI, .06–.37] for N+WSH). There was no difference in total pathogens—nor when broken down by bacteria, viruses, or parasites—between the combined N+WSH intervention and either the WSH- or nutrition-only arm. Virus results were robust across unadjusted and adjusted models (Supplementary Figure 4*A*).

**Table 2.** Differences in the Number of Co-occurring Pathogens Between Intervention Arms in Fecal Samples Collected From Rural Bangladeshi Children Aged 14 Months Enrolled in the WASH Benefits Randomized Controlled Trial Environmental Enteric Dysfunction Substudy

Pathogen Group, Study Arm	No. of Co-occurring Pathogens						Pathogen Difference^b^ (95% CI)	
	Total^a^		Monsoon Season		Dry Season			
	No.	Mean (SD)	No.	Mean (SD)	No.	Mean (SD)	Intervention vs Control	N+WSH vs Intervention
All pathogens								
Control	299	3.8 (1.80)	116	3.8 (1.69)	183	3.9 (1.87)	NA	NA
WSH	351	3.8 (1.72)	266	4.1 (1.66)	84	2.9 (1.61)	**–0.50 (–.90 to –.07)**	0.17 (–.05 to .52)
Nutrition	344	4.0 (1.84)	233	4.3 (1.80)	111	3.4 (1.80)	–0.19 (–.68 to .18)	–0.02 (–.38 to .27)
N+WSH	339	4.1 (1.89)	251	4.3 (1.91)	88	3.4 (1.67)	–0.16 (–.65 to .17)	NA
Bacteria								
Control	323	2.80 (1.38)	121	2.8 (1.34)	202	2.8 (1.41)	NA	NA
WSH	363	3.0 (1.53)	273	3.3 (1.49)	89	2.2 (1.33)	–0.18 (–.50 to .10)	0.08 (–.11 to .35)
Nutrition	348	3.1 (1.57)	236	3.4 (1.60)	112	2.4 (1.31)	–0.07 (–.42 to .15)	0.02 (–.25 to .32)
N+WSH	352	3.1 (1.56)	257	3.4 (1.60)	95	2.5 (1.26)	–0.04 (–.35 to .22)	NA
Viruses								
Control	312	0.60 (0.63)	123	0.60 (0.70)	189	0.60 (0.58)	NA	NA
WSH	358	0.30 (0.56)	271	0.30 (0.56)	86	0.40 (0.55)	**–0.28 (–.48 to –.09)**	0.15 (–.02 to .24)
Nutrition	349	0.40 (0.64)	237	0.40 (0.59)	112	0.50 (0.73)	**–0.17 (–.38 to –.05)**	–0.02 (–.16 to .10)
N+WSH	349	0.40 (0.63)	258	0.40 (0.60)	91	0.50 (0.69)	**–0.21 (–.37 to –.06)**	NA
Parasites								
Control	315	0.40 (0.65)	118	0.30 (0.58)	197	0.40 (0.68)	NA	NA
WSH	358	0.40 (0.65)	270	0.50 (0.67)	87	0.30 (0.55)	–0.01 (–.17 to .09)	0.01 (–.06 to .17)
Nutrition	347	0.50 (0.78)	234	0.50 (0.80)	113	0.50 (0.70)	0.03 (–.06 to .28)	–0.01 (–.21 to .09)
N+WSH	346	0.50 (0.70)	254	0.50 (0.70)	92	0.40 (0.71)	0.02 (–.09 to .20)	NA

Bold font indicates significance.

Abbreviations: CI, confidence interval; NA, not applicable; N+WSH, nutrition plus water, sanitation, and handwashing; WSH, water, sanitation, and handwashing.

^a^Includes both monsoon (May–October) and dry (November–April) seasons.

^b^Adjusted for prespecified covariates using generalized linear models and g-computation estimator: household food insecurity, child age, child sex, child birth order, season of sample collection, time between defecation and sample placed on cold chain, mother’s age, mother’s height, mother’s education level, number of children <18 years of age in the household, number of individuals living in the compound, distance in minutes to the primary water source, household floor and wall materials, household assets.

In control children, the stunting-associated pathogen composite score (including EAEC, *Shigella*/EIEC, *Campylobacter* spp, and *Giardia* spp) was associated with concurrent, but not future, length-for-age *z* score (Supplementary Figure 5). There was no difference in the total load of the stunting-associated pathogens between children in any of the intervention arms compared with controls, nor between N+WSH children compared with children receiving single WSH or nutrition interventions (Supplementary Figure 4*B*).

### Impact of Interventions on Individual Pathogen Prevalence and Quantity

**Figure 1.**
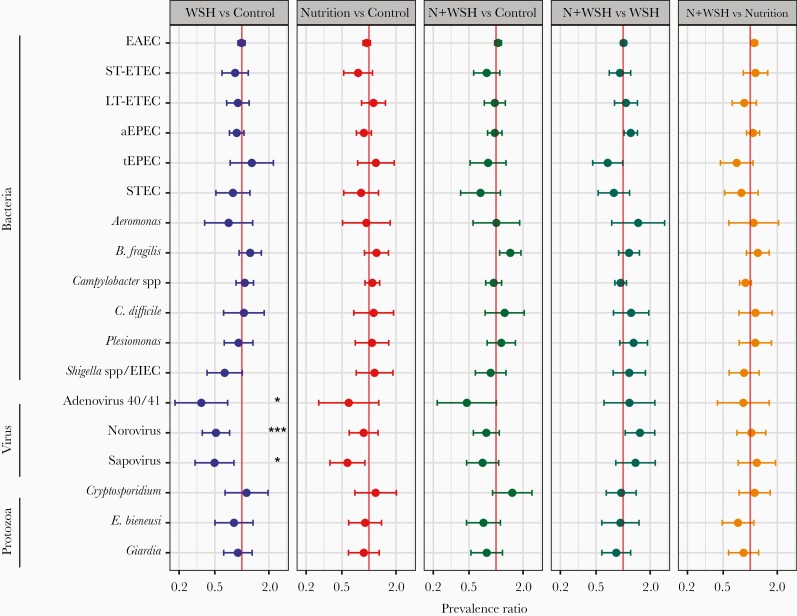
Impact of interventions on prevalence ratio of individual pathogens in 14-month-old children from rural Bangladesh. Point estimates and 95% confidence intervals were determined with a generalized linear model adjusting for covariates associated with each pathogen outcome (likelihood ratio test *P* < .1 in bivariate analysis): household food insecurity, child age, child sex, child birth order, season of sample collection, time between defecation and sample placed on cold chain, mother’s age, mother’s height, mother’s education level, number of children aged <18 years in the household, number of individuals living in the compound, distance in minutes to the primary water source, household floor and wall materials, and household assets. Pathogens significant after correction for false discovery rate are annotated: *P < .05, ***P < .005. Abbreviations: aEPEC, atypical enteropathogenic *Escherichia coli*; EAEC, enteroaggregative *Escherichia coli*; EIEC, enteroinvasive *Escherichia coli*; LT-ETEC, enterotoxigenic *Escherichia coli* with heat-labile toxin; N+WSH, nutrition plus water, sanitation, and handwashing; STEC, Shiga toxin–producing *Escherichia coli*; ST-ETEC, enterotoxigenic *Escherichia coli* with heat-stable toxin; tEPEC, typical enteropathogenic *Escherichia coli*; WSH, water, sanitation, and handwashing.

**Figure 2.**
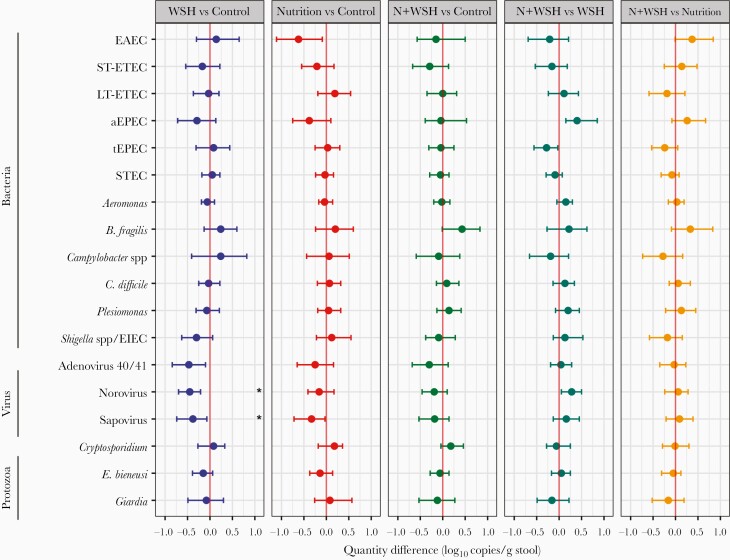
Impact of interventions on quantity (log_10_ copies/gram stool) of individual pathogens in 14-month-old rural Bangladeshi children. Point estimates and 95% confidence intervals determined with a parametric g-formula including both logistic and log-linear regression steps with generalized linear models adjusting for covariates associated with each pathogen outcome (likelihood ratio test *P* < .1 in bivariate analysis): household food insecurity, child age, child sex, child birth order, season of sample collection, time between defecation and sample placed on cold chain, mother’s age, mother’s height, mother’s education level, number of children aged <18 years in the household, number of individuals living in the compound, distance in minutes to the primary water source, household floor and wall materials, and household assets. Pathogens significant after correction for false discovery rate are annotated: *P < .05. Abbreviations: aEPEC, atypical enteropathogenic *Escherichia coli*; EAEC, enteroaggregative *Escherichia coli*; EIEC, enteroinvasive *Escherichia coli*; LT-ETEC, enterotoxigenic *Escherichia coli* with heat-labile toxin; N+WSH, nutrition plus water, sanitation, and handwashing; STEC, Shiga toxin–producing *Escherichia coli*; ST-ETEC, enterotoxigenic *Escherichia coli* with heat-stable toxin; tEPEC, typical enteropathogenic *Escherichia coli*; WSH, water, sanitation, and handwashing.

Compared with controls, children receiving WSH interventions had significantly lower prevalence of norovirus, sapovirus, and adenovirus 40/41 (Figure 1 for prevalence ratios, Table 3 for prevalence differences). This corresponded to a mean 0.45 (95% CI, .21–.70) log_10_ fewer norovirus, 0.38 (95% CI, .07–.74) log_10_ fewer sapovirus, and 0.47 (95% CI, .10–.84) log_10_ fewer adenovirus 40/41 when evaluated quantitatively (Figure 2 and Supplementary Table 4). These findings were consistent with unadjusted estimates. Children receiving the nutrition-only intervention had an absolute 8%–10% lower prevalence of EAEC and sapovirus than controls (Table 3), with similar results obtained for pathogen quantity (0.62 [95% CI, .09–1.11] log_10_ fewer EAEC; 0.33 [95% CI, .03–.72] log_10_ fewer sapovirus; Figure 2 and Supplementary Table 4), but neither was significant after multiple comparisons correction.

**Table 3.** Prevalence Difference Between Intervention Arms for Individual Pathogens

Pathogen and Arm	Pathogen Prevalence						Prevalence Difference Between Arms^a^			
	Total^b^		Monsoon Season		Dry Season		Intervention vs Control		N+WSH vs Intervention	
	No.	Mean (SD)	No.	Mean (SD)	No.	Mean (SD)	PD (95% CI)	*P* Value^c^	PD (95% CI)	*P* Value^c^
EAEC										
Control	328	0.76 (0.43)	123	0.8 (0.4)	205	0.73 (0.44)	NA	NA	NA	NA
WSH	364	0.79 (0.41)	274	0.83 (0.38)	89	0.69 (0.47)	–0.01 (–.08 to .06)	.84	0.01 (–.05 to .06)	.83
Nutrition	350	0.72 (0.45)	237	0.75 (0.43)	113	0.65 (0.48)	–0.1 (–.17 to –.02)	.10	0.08 (.01–.14)	.29
N+WSH	354	0.8 (0.4)	259	0.81 (0.39)	95	0.76 (0.43)	0 (–.08 to .08)	1.00	NA	NA
ST-ETEC										
Control	328	0.18 (0.38)	123	0.24 (0.43)	205	0.14 (0.34)	NA	NA	NA	NA
WSH	364	0.22 (0.42)	274	0.27 (0.45)	89	0.07 (0.25)	–0.02 (–.09 to .04)	.67	–0.01 (–.07 to .05)	.81
Nutrition	350	0.18 (0.38)	237	0.24 (0.43)	113	0.06 (0.24)	–0.05 (–.11 to .02)	.65	0.03 (–.03 to .09)	.65
N+WSH	354	0.21 (0.41)	259	0.27 (0.44)	95	0.04 (0.2)	–0.04 (–.1 to .03)	.54	NA	NA
LT-ETEC										
Control	326	0.19 (0.39)	123	0.17 (0.38)	203	0.2 (0.4)	NA	NA	NA	NA
WSH	364	0.19 (0.39)	274	0.22 (0.42)	89	0.08 (0.27)	–0.04 (–.1 to .01)	.42	0.02 (–.04 to .07)	.81
Nutrition	350	0.23 (0.42)	237	0.26 (0.44)	113	0.18 (0.38)	0.02 (–.04 to .09)	.65	–0.03 (–.1 to .04)	.65
N+WSH	354	0.21 (0.41)	259	0.23 (0.42)	95	0.16 (0.37)	0 (–.06 to .05)	1.00	NA	NA
aEPEC										
Control	327	0.46 (0.50)	122	0.38 (0.49)	205	0.51 (0.50)	NA	NA	NA	NA
WSH	364	0.36 (0.48)	274	0.34 (0.47)	89	0.43 (0.5)	–0.05 (–.13 to .02)	.47	0.08 (.01–.15)	.26
Nutrition	350	0.4 (0.49)	237	0.4 (0.49)	113	0.41 (0.49)	–0.06 (–.14 to .02)	.65	0.04 (–.03 to .11)	.65
N+WSH	353	0.44 (0.5)	258	0.41 (0.49)	95	0.51 (0.5)	0 (–.09 to .08)	1.00	NA	NA
tEPEC										
Control	328	0.08 (0.28)	123	0.11 (0.32)	205	0.06 (0.24)	NA	NA	NA	NA
WSH	364	0.15 (0.36)	274	0.17 (0.38)	89	0.08 (0.27)	0.03 (–.02 to .08)	.49	–0.05 (–.09 to 0)	.35
Nutrition	350	0.13 (0.34)	237	0.19 (0.39)	113	0.03 (0.16)	0.02 (–.03 to .07)	.65	–0.04 (–.09 to .01)	.58
N+WSH	353	0.1 (0.3)	258	0.14 (0.34)	95	0.01 (0.1)	–0.01 (–.05 to .03)	.79	NA	NA
STEC										
Control	327	0.11 (0.31)	122	0.18 (0.39)	205	0.07 (0.25)	NA	NA	NA	NA
WSH	364	0.13 (0.34)	274	0.14 (0.35)	89	0.09 (0.29)	–0.02 (–.07 to .03)	.67	–0.03 (–.07 to .02)	.66
Nutrition	350	0.12 (0.32)	237	0.14 (0.35)	113	0.07 (0.26)	–0.03 (–.08 to .03)	.65	–0.02 (–.07 to .02)	.65
N+WSH	354	0.1 (0.3)	259	0.11 (0.31)	95	0.08 (0.28)	–0.04 (–.1 to .01)	.46	NA	NA
*Aeromonas*										
Control	327	0.06 (0.25)	123	0.03 (0.18)	204	0.08 (0.28)	NA	NA	NA	NA
WSH	364	0.05 (0.22)	274	0.07 (0.25)	89	0 (0)	–0.02 (–.06 to .01)	.49	0.02 (–.02 to .06)	.66
Nutrition	350	0.06 (0.24)	237	0.08 (0.27)	113	0.03 (0.16)	–0.01 (–.04 to .03)	.75	0.01 (–.03 to .05)	.77
N+WSH	354	0.07 (0.25)	259	0.09 (0.29)	95	0.01 (0.1)	0 (–.04 to .04)	1.00	NA	NA
*Bacteroides fragilis*										
Control	328	0.19 (0.39)	123	0.14 (0.35)	205	0.21 (0.41)	NA	NA	NA	NA
WSH	364	0.23 (0.42)	274	0.24 (0.43)	89	0.2 (0.4)	0.04 (–.01 to .1)	.42	0.04 (–.03 to .11)	.66
Nutrition	350	0.23 (0.42)	237	0.23 (0.42)	113	0.21 (0.41)	0.04 (–.02 to .1)	.65	0.05 (–.02 to .12)	.65
N+WSH	354	0.28 (0.45)	259	0.29 (0.45)	95	0.25 (0.44)	0.08 (.02–.15)	.18	NA	NA
*Campylobacter* spp										
Control	328	0.41 (0.49)	123	0.39 (0.49)	205	0.43 (0.5)	NA	NA	NA	NA
WSH	364	0.49 (0.5)	274	0.53 (0.5)	89	0.36 (0.48)	0.04 (–.06 to .13)	.67	–0.03 (–.1 to .04)	.66
Nutrition	350	0.52 (0.5)	237	0.56 (0.5)	113	0.42 (0.5)	0.04 (–.04 to .12)	.65	–0.07 (–.14 to .01)	.58
N+WSH	354	0.47 (0.5)	259	0.52 (0.5)	95	0.34 (0.48)	–0.03 (–.11 to .06)	.79	NA	NA
*Clostridioides difficile*										
Control	328	0.08 (0.28)	123	0.08 (0.27)	205	0.08 (0.28)	NA	NA	NA	NA
WSH	363	0.09 (0.28)	273	0.1 (0.29)	89	0.07 (0.25)	0 (–.04 to .05)	.84	0.02 (–.02 to .06)	.66
Nutrition	348	0.09 (0.29)	236	0.08 (0.28)	112	0.12 (0.32)	0.01 (–.03 to .06)	.71	0.02 (–.03 to .06)	.66
N+WSH	353	0.11 (0.31)	258	0.1 (0.3)	95	0.14 (0.35)	0.02 (–.03 to .07)	.67	NA	NA
*Plesiomonas*										
Control	328	0.14 (0.34)	123	0.1 (0.3)	205	0.16 (0.37)	NA	NA	NA	NA
WSH	363	0.15 (0.35)	273	0.18 (0.39)	89	0.03 (0.18)	–0.01 (–.06 to .04)	.69	0.04 (–.01 to .1)	.65
Nutrition	348	0.16 (0.36)	236	0.19 (0.39)	112	0.08 (0.27)	0.01 (–.05 to .07)	.75	0.02 (–.05 to .09)	.66
N+WSH	353	0.18 (0.39)	258	0.2 (0.4)	95	0.15 (0.36)	0.02 (–.04 to .08)	.75	NA	NA
*Shigella* spp/EIEC										
Control	328	0.13 (0.33)	123	0.15 (0.36)	205	0.11 (0.31)	NA	NA	NA	NA
WSH	364	0.13 (0.34)	274	0.16 (0.37)	89	0.03 (0.18)	–0.04 (–.1 to .01)	.42	0.02 (–.04 to .08)	.69
Nutrition	350	0.17 (0.37)	237	0.19 (0.4)	113	0.12 (0.32)	0.02 (–.04 to .09)	.65	–0.02 (–.09 to .04)	.66
N+WSH	354	0.15 (0.36)	259	0.19 (0.39)	95	0.05 (0.22)	–0.01 (–.07 to .04)	.79	NA	NA
Adenovirus 40/41										
Control	328	0.1 (0.31)	123	0.22 (0.42)	205	0.03 (0.18)	NA	NA	NA	NA
WSH	364	0.06 (0.23)	274	0.07 (0.25)	89	0.03 (0.18)	–0.09 (–.15 to –.02)	.08	0.01 (–.03 to .05)	.81
Nutrition	350	0.08 (0.28)	237	0.08 (0.28)	113	0.08 (0.27)	–0.05 (–.12 to .03)	.65	–0.01 (–.06 to .04)	.68
N+WSH	354	0.07 (0.26)	259	0.07 (0.25)	95	0.08 (0.28)	–0.06 (–.14 to .01)	.46	NA	NA
Norovirus										
Control	329	0.23 (0.42)	124	0.21 (0.41)	205	0.24 (0.43)	NA	NA	NA	NA
WSH	369	0.11 (0.32)	274	0.13 (0.33)	94	0.07 (0.26)	**0.11 (–.17 to –.05)**	**.004**	0.06 (.01–.12)	.26
Nutrition	353	0.18 (0.38)	237	0.14 (0.35)	116	0.25 (0.43)	–0.03 (–.1 to .05)	.65	0.01 (–.06 to .07)	.88
N+WSH	360	0.18 (0.38)	260	0.18 (0.38)	100	0.18 (0.39)	–0.05 (–.12 to .02)	.46	NA	NA
Sapovirus										
Control	313	0.19 (0.4)	124	0.15 (0.35)	189	0.23 (0.42)	NA	NA	NA	NA
WSH	364	0.09 (0.28)	272	0.08 (0.27)	91	0.1 (0.3)	**–0.09 (–.15 to –.03)**	**.03**	0.03 (–.02 to .08)	.66
Nutrition	352	0.1 (0.3)	237	0.09 (0.29)	115	0.12 (0.33)	–0.08 (–.14 to –.02)	.10	0.02 (–.03 to .07)	.66
N+WSH	355	0.12 (0.32)	259	0.1 (0.3)	96	0.17 (0.37)	–0.05 (–.12 to .01)	.46	NA	NA
*Cryptosporidium*										
Control	328	0.08 (0.27)	123	0.09 (0.29)	205	0.07 (0.26)	NA	NA	NA	NA
WSH	364	0.15 (0.35)	274	0.17 (0.38)	89	0.07 (0.25)	0.02 (–.03 to .07)	.67	–0.01 (–.06 to .05)	.83
Nutrition	350	0.12 (0.33)	237	0.14 (0.35)	113	0.09 (0.29)	0.02 (–.03 to .07)	.65	0.01 (–.04 to .07)	.68
N+WSH	354	0.14 (0.35)	259	0.14 (0.35)	95	0.14 (0.35)	0.04 (–.01 to .1)	.46	NA	NA
*Enterocytozoon bieneusi*										
Control	327	0.11 (0.31)	123	0.09 (0.29)	204	0.12 (0.32)	NA	NA	NA	NA
WSH	364	0.1 (0.3)	274	0.11 (0.31)	89	0.08 (0.27)	–0.02 (–.07 to .03)	.67	–0.01 (–.05 to .04)	.83
Nutrition	350	0.12 (0.32)	237	0.14 (0.34)	113	0.08 (0.27)	–0.01 (–.06 to .04)	.70	–0.03 (–.08 to .01)	.58
N+WSH	354	0.09 (0.29)	259	0.1 (0.3)	95	0.06 (0.24)	–0.03 (–.07 to .01)	.47	NA	NA
*Giardia*										
Control	328	0.14 (0.34)	123	0.07 (0.26)	205	0.18 (0.38)	NA	NA	NA	NA
WSH	364	0.15 (0.35)	274	0.15 (0.36)	89	0.12 (0.33)	–0.01 (–.07 to .04)	.69	–0.02 (–.07 to .03)	.66
Nutrition	350	0.14 (0.34)	237	0.12 (0.33)	113	0.17 (0.38)	–0.02 (–.07 to .04)	.65	–0.02 (–.07 to .03)	.65
N+WSH	354	0.13 (0.33)	259	0.13 (0.34)	95	0.12 (0.32)	–0.03 (–.08 to .02)	.53	NA	NA

Fecal samples (n = 1411) were collected from rural Bangladeshi children aged 14 months enrolled in the WASH Benefits randomized controlled trial environmental enteric dysfunction substudy. Bold font indicates significance after correcting for false discovery rate using the Benjamini–Hochberg procedure.

Abbreviations: aEPEC, atypical enteropathogenic *Escherichia coli*; CI, confidence interval; EAEC, enteroaggregative *Escherichia coli*; EIEC, enteroinvasive *Escherichia coli*; LT-ETEC, enterotoxigenic *Escherichia coli* with heat-labile toxin; NA, not applicable; N+WSH, nutrition plus water, sanitation, and handwashing; PD, prevalence difference; SD, standard deviation; STEC, Shiga toxin–producing *Escherichia coli*; ST-ETEC, enterotoxigenic *Escherichia coli* with heat-stable toxin; tEPEC, typical enteropathogenic *Escherichia coli*; WSH, water, sanitation, and handwashing.

^a^Adjusted for prespecified covariates with a likelihood ratio test *P* < .1 in bivariate analysis with the outcome: household food insecurity; child age, sex, and birth order; season of sample collection; time until sample placed on cold chain; mother’s age, height, and education level; number of children <18 years of age in the household; number of individuals living in the compound; distance in minutes to the primary water source; household floor and wall materials; household assets.

^b^Includes both monsoon (May–October) and dry (November–April) seasons.

^c^ *P* values shown are adjusted for false discovery rate using the Benjamini–Hochberg procedure.

There was no difference in the prevalence or quantity of individual pathogens when comparing N+WSH children to those from the single nutrition arm, but there was a 6% (95% CI, 1%–12%) higher prevalence and 0.28 (95% CI, .05–.50) higher log_10_ quantity of norovirus compared with children receiving the WSH-only intervention, although neither was significant after multiple comparisons correction (Figures 1 and 2, Table 3, and Supplementary Table 4).

Notably, there were no differences in protozoa between study arms. Results across all outcomes were comparable when adjusting for missing outcomes (children who did not provide fecal samples), when adjusting only for child age and season of sample collection, or when evaluating only samples from children with no reported diarrheal symptoms within the past 7 days (Supplementary Tables 5 and 6 and Supplementary Figure 4). Seasonal effect modification of intervention efficacy was not observed; however, point estimates suggested slightly greater efficacy during the dry season for some pathogens (Supplementary Figures 6–8).

## DISCUSSION

Children in rural Bangladesh receiving low-cost WSH, nutrition, or N+WSH interventions [38, 39] had similar prevalence and quantity of bacterial and parasitic enteropathogens in their stool at 14 months old compared with controls. Importantly, the 4 bacterial/protozoan enteropathogens most commonly associated with stunting [5] were not impacted by the interventions, both when evaluated individually or in total. This is consistent with lack of effect of the WSH interventions on child growth in the main trial, and a modest effect of nutrition interventions that was equivalent to other nutrition-only trials [22, 40]. However, the WSH intervention was associated with fewer enteric viruses, as evidenced by lower total viruses and lower prevalence and/or log_10_ quantity of norovirus, sapovirus, and adenovirus 40/41. Viruses are among the top diarrhea-causing pathogens in this age group [27, 28], and the combined reduction in viruses (~30%) is consistent with the diarrhea reductions reported for children whose households received WSH interventions [22], providing strong evidence for causality. Despite similar pathogen carriage profiles, children receiving nutrition interventions had lower diarrhea prevalence than controls [22], suggesting the nutrition intervention might be preventing illness when children are exposed to enteropathogens. Some parasite results were discordant with those from the main trial when children were older, which is discussed below.

Our findings parallel those of the SHINE trial in rural Zimbabwe. Both trials found no impact of nutrition interventions on enteropathogens [13]. The SHINE trial found that WSH interventions reduced *neither* diarrhea nor enteropathogens for children 6–12 months old [13, 41], whereas our study found *both* lower diarrhea *and* lower viral pathogens for children 14 months old receiving WSH interventions compared with controls. Less frequent intervention promotion could have led to lower adherence in the SHINE trial [23].

Our nutritional intervention was not associated with increased diarrhea or enteropathogens. Importantly, our supplement was consumed twice daily (4.5 mg iron per dose) to increase host absorption and reduce bacterial iron scavenging, compared with micronutrient powders that supply 1 daily 12.5-mg iron dose [17]. Furthermore, children receiving the nutrition intervention had less transferrin receptor [32], one viral mechanism for cellular invasion [42], possibly explaining the lower total viruses for this group. Although nonsignificant after multiple hypothesis correction, norovirus prevalence and quantity were higher in the N+WSH arm than the WSH arm, suggesting a possible interaction between the nutrition and WSH interventions. A potential explanation is that the nutrition intervention increased specific populations of gut bacteria, resulting in improved norovirus survivability and infectivity [43]. This is not at odds with the growth outcomes from the main trial as viruses are not the top pathogen predictors of stunting.

An important limitation of this study was imbalance in child age and season between study arms at the time of fecal sample collection. Political unrest associated with the Bangladesh 2014 general election disproportionately impacted our ability to collect samples in control villages, where community members were not familiar with the project due to lack of visible interventions or routine visits by health promoters. Imbalance in age between arms is important because pathogen prevalence increases over the first 2 years of life [5]; control children from our substudy were already walking at the time of sample collection (study children began walking at 13.0 [IQR, 11.9–14.0] months [29]), potentially increasing their environmental exposure to pathogens. The observed seasonal variability of enteropathogens is consistent with previous data from Bangladesh and seasonal diarrhea patterns from the WASH Benefits trial [2, 22], which could have biased our findings toward the null. To address these limitations, we relied on adjusted analyses that controlled for these covariates. We found no effect modification of intervention effectiveness by season, but estimates were imprecise due to low power to detect interaction.

Another limitation was the use of a single assessment to measure the impact of interventions on enteropathogens. Incidence of enteric viruses peaks at the age we evaluated [27]; however, the median age at initial detection of *Giardia* in surveillance stools in Bangladesh was 18 months [44]. Persistent *Giardia* and helminth infections are common, increasing the proportion of infected children at older ages; thus, cumulative differences would increase with a successful intervention as children age [44]. In contrast to our observations in 14-month-old children, those who received WSH and N+WSH interventions had a reduced prevalence of *Giardia* and hookworm at age 30 months [24, 25]. These former analyses had greater statistical power from a 50% larger sample size and geographic block–matched analysis. Thus, we cautiously interpret our parasite results: The young age at which we measured was not ideal to evaluate protozoa/helminths, and smaller sample size and covariate imbalances weakened our ability to detect differences.

Notably, our strongest findings were for enteric viruses, the pathogens least influenced by season and child age in this study. Estimated differences in virus quantities were small in comparison to fecal loads (difference of approximately 0.5 of 5–7 log_10_ copies/g stool). However, viruses are excreted at orders of magnitude higher per gram of feces and have low infectious doses (10–1000 viral particles) [45]. Thus, interventions that disrupt transmission of all types of enteropathogens might see greater effects in enteric viruses compared with bacteria/parasites with similar infectious doses and even greater effects than those with higher infectious doses. The prevalence of norovirus, sapovirus, and adenovirus 40/41 were each approximately 10% lower in WSH children than controls, and these pathogens account for 25% of all diarrhea cases for children <24 months old in Bangladesh [27, 28]. This suggests that WSH interventions can be clinically relevant for diarrheal disease. Our adjusted and unadjusted analyses for viruses led to the same scientific inference, and our data are consistent with the reductions in diarrhea reported in the main trial for the WSH intervention, which gives us high confidence that WSH interventions reduced enteric viruses in 14-month-old children.

Rotavirus prevalence in our study was similar to previous cross-sectional studies where most children do not have diarrhea [46]. However, the prevalence in children with diarrhea was low for Bangladesh prior to implementation of rotavirus vaccination [27], likely because our sample collection period (February–November) did not include peak rotavirus season (December–January) [47].

There is no evidence for differences in efficacy of WSH interventions against bacteria compared with viruses. Handwashing with soap had similar removal efficacy for both, chlorine inactivation is comparable, and no difference in decay rates for bacteria and viruses have been observed on foods or household inanimate objects [48–50]. The evidence that WSH interventions impacted viruses is stronger than for bacteria given the influence of seasonality on some bacterial pathogens. Thus, we are less confident that our results indicate definitively that WSH and nutrition interventions did not impact bacterial pathogens.

WSH interventions were associated with lower enteric viruses in children 14 months old compared with controls, which is a modest impact on overall enteropathogens. These viruses account for a quarter of the diarrheal episodes for children <2 years old in Bangladesh, indicating a potentially clinically meaningful impact on childhood diarrhea. Our results suggest that neither low-cost household-level WSH nor nutrition interventions are sufficient to disrupt nonviral enteropathogen carriage at this critical early age. This is consistent with the lack of effect of WSH interventions on growth as the primary stunting-associated pathogens are thought to be bacterial and protozoan. Future interventions should be designed with consideration for transmission pathways and environmental or zoonotic reservoirs of bacterial and parasitic enteropathogens to maximize efficacy for improving child health beyond diarrheal disease to include ponderal growth and cognitive development.

## Supplementary Data

Supplementary materials are available at *The Journal of Infectious Diseases online*. Consisting of data provided by the authors to benefit the reader, the posted materials are not copyedited and are the sole responsibility of the authors, so questions or comments should be addressed to the corresponding author.

jiaa549_suppl_Supplementary_Material

## Notes

***Author contributions***. J. A. G. led the analysis and drafted the manuscript with input from coauthors. M. R., L. U., S. P. L., C. P. S., J. M. C., B. F. A., J. B.-C., and A. L. helped develop interventions and oversaw trial implementation. S. A., S. L. F., M. S. H., P. M., Z. H., M. Z. R., A. K. S., A. L., and J. A. G. were responsible for field sample and data collection/management. M. A. K., R. M., M. O. I., J. L., M. T., R. H., and J. A. G. contributed to pathogen data acquisition. J. A. G., E. T. R. M., B. F. A., A. L., J. B.-C., J. A. P.-M., S. P. H., J. L., and S. P. L. contributed to data analysis and interpretation. S. P. L., J. A. G., and E. R. H. conceived of the study.

***Acknowledgments***. We gratefully acknowledge the study participants and their families. We thank the intervention delivery, sample collection, and laboratory teams at icddr,b for their tremendous dedication in conducting high-quality fieldwork, including during periods of civil unrest.

***Data sharing***. The WASH Benefits trial protocol was previously published [30], and the statistical analysis plan, de-identified data, and analysis files for this study are published online at: https://osf.io/ky275/.

***Disclaimer***. The funders had no role in study design, data collection and analysis, decision to publish, or preparation of the manuscript.

***Financial support***. This work was supported by the Bill & Melinda Gates Foundation (award numbers OPPGD759 and OPP1161946 for project funds, and OPP1179069 to J. A. P.-M.); the National Science Foundation (graduate research fellowship to J. A. G.); Stanford University (Stanford Interdisciplinary Graduate Fellowship to J. A. G.); and the National Institute of Allergy and Infectious Diseases, National Institutes of Health (grant number K01AI130326 to E. T. R. M.).

***Potential conflicts of interest***. All authors: No reported conflicts of interest.
