# Supplementary material for: Effect of Water, Sanitation, Handwashing, and Nutrition Interventions on Enteropathogens in Children 14 Months Old: A Cluster-Randomized Controlled Trial in Rural Bangladesh
Source: J Infect Dis. 2020 Aug 29;227(3):434–47. doi: 10.1093/infdis/jiaa549 (PMC9891429; doi:10.1093/infdis/jiaa549)
Supplement: jiaa549_suppl_Supplementary_Material [file jiaa549_suppl_supplementary_material.pdf]

Effects of water, sanitation, handwashing and nutrition interventions on enteropathogens in children 14 months old: a cluster-randomised controlled trial in rural Bangladesh

Supplemental Materials

Jessica A. Grembi[1], Audrie Lin[2], Md. Abdul Karim[3], Md. Ohedul Islam[3], Rana Miah[3], Benjamin F. Arnold[4], Elizabeth T. Rogawski McQuade[5], Shahjahan Ali[3], Md. Ziaur Rahman[3], Zahir Hussain[3], Abul K. Shoab[3], Syeda L. Famida[3], Md. Saheen Hossen[3], Palash Mutsuddi[3], Mahbubur Rahman[3], Leanne Unicomb[3], Rashidul Haque[3], Mami Taniuchi[5], Jie Liu[5], James A. Platts-Mills[5], Susan P. Holmes[6], Christine P. Stewart[7], Jade Benjamin-Chung[2], John M. Colford Jr.[2], Eric R. Houpt[5], and Stephen P. Luby[1]

[1]Division of Infectious Diseases and Geographic Medicine, Stanford University, Stanford, CA USA
[2]Division of Epidemiology and Biostatistics, School of Public Health, University of California, Berkeley, Berkeley, CA USA
[3]Infectious Diseases Division, International Centre for Diarrheal Disease Research, Bangladesh, Dhaka, Bangladesh
[4]Francis I. Proctor Foundation, University of California, San Francisco, San Francisco, CA USA
[5]Division of Infectious Diseases International Health, University of Virginia, Charlottesville, VA USA
[6]Department of Statistics, Stanford University, Stanford, CA USA
[7]Institute for Global Nutrition, University of California, Davis, Davis, CA USA

July 2020

Table S1: Real time PCR assays on the TaqMan Array Card and associated gene targets. Assays have been described previously and extensively validated.[1–3] Nucleic acid was extracted with the QIAamp Fast DNA Stool mini kit (Qiagen, Hilden, Germany) with pre-treatment steps that included bead beating. AgPath One Step RT-PCR reagents were used for qPCR reactions, which were performed on ViiA 7 systems. Quantification cycles (Cqs) are the PCR cycle values at which fluorescence from amplification exceeds the background, which acts as an inverse metric of quantity of nucleic acid. Valid results required proper functioning of controls (the negative results of a sample are valid only when its external control MS2 is positive, Cq 35; the positive results are valid only when the corresponding extraction blank is negative for the relevant targets, Cq ¿ 35), and excluded data flagged by the real time PCR software, i.e. BADROX in combination with NOISE or SPIKE.

Pathogen	Gene
**Virus**	
Adenovirus 40/41	Fiber gene
Astrovirus	Capsid
Norovirus GI/GII	GI ORF1-2 and GII ORF1-2
Rotavirus	*NSP3*
Sapovirus	*RdRp*
**Bacteria**	
Enteroaggregative *Escherichia coli* (EAEC)*	*aaiC*, *aatA*
Enteropathogenic *E. coli* (EPEC)*	*bfpA*, *eae*
Enterotoxigenic *E. coli* (ETEC)*	*LT*, *STh*, *STp*
Shiga toxin-producing *E. coli* (STEC)*	*stx1*, *stx2*
Aeromonas	Aerolysin
Bacteroides fragilis	EGBF
Campylobacter spp.	*cpn60*
Campylobacter jejuni/coli	*cadF*
Clostridium difficile	*tcdA*, *tcdB*
Helicobacter pylori	*ureC*
Plesiomonas shigelloides	*gyrB*
Salmonella enterica	*ttr*
Shigella spp./Enteroinvasive *E. coli* (EIEC)	*ipaH*
Vibrio cholerae	*hlyA*
**Fungi**	
Encephalitozoon intestinalis	SSU rRNA
Enterocytozoon bieneusi	*ITS*
**Protozoa**	
Cryptosporidium spp.	18S rRNA
Entamoeba histolytica	18S rRNA
Entamoeba spp.	18S rRNA
Giardia spp.	18S rRNA
Cyclospora cayetanensis	18S rRNA
Cystoisospora belli	18S rRNA
**Helminth**	
Ancylostoma duodenale	*ITS2*
Ascaris lumbricoides	*ITS1*
Blastocystis spp.	18S rRNA
Hymenolepis nana	*ITS1*
Necator americanus	*ITS2*
Strongyloides stercoralis	Dispersed repetitive sequence
Schistosoma spp.	*ITS*
Trichuris trichiura	18S rRNA
**Controls**	
MS2	*MS2g1*
PhHV	*gB*

* *E. coli* pathotypes were defined as follows: EAEC (*aaiC*, or *aatA*, or both), atypical EPEC (*eae* without *bfpA*, *stx1*, and *stx2*), typical EPEC (*bfpA* and *eae*), ETEC (STh, STp, or LT), STEC (*eae* without *bfpA* and with *stx1*, *stx2*, or both).

Table S2: Household enrollment characteristics between EE cohort (this study) and main trial cohort.

	Control	WSH	Nutrition	WSH+N	Control	WSH	Nutrition	WSH+N
	EE Cohort				Main Trial Cohort			
	(n = 449)	(n = 446)	(n = 435)	(n = 447)	(n = 1382)	(n = 702)	(n = 699)	(n = 686)
Enrollment Characteristics								
**Maternal**								
Age (years)	23 (5)	24 (5)	24 (5)	24 (5)	24 (5)	24 (5)	24 (5)	24 (6)
Height (cm)	151 (5)	150 (5)	150 (6)	150 (5)	151 (5)	151 (5)	150 (5)	150 (5)
Maternal Years of education	7 (3)	6 (3)	6 (4)	6 (3)	6 (3)	6 (3)	6 (3)	6 (3)
**Paternal**								
Paternal Years of Education	5 (4)	5 (4)	5 (4)	5 (4)	5 (4)	5 (4)	5 (4)	5 (4)
Works in agriculture	104 (23%)	128 (29%)	148 (34%)	127 (28%)	414 (30%)	216 (31%)	232 (33%)	207 (30%)
**Household**								
Number of people	5 (2)	5 (2)	5 (2)	5 (2)	5 (2)	5 (2)	5 (2)	5 (2)
Number of children under 18 years	2 (1)	2 (1)	2 (1)	2 (1)	2 (1)	2 (1)	2 (1)	2 (1)
Has electricity	269 (60%)	278 (62%)	269 (62%)	272 (61%)	784 (57%)	426 (61%)	409 (59%)	412 (60%)
Has a cement floor	75 (17%)	55 (12%)	50 (11%)	53 (12%)	145 (10%)	77 (11%)	67 (10%)	72 (10%)
Acres of agricultural land owned	0.18 (0.25)	0.17 (0.26)	0.17 (0.3)	0.13 (0.18)	0.15 (0.21)	0.15 (0.23)	0.16 (0.27)	0.14 (0.38)
**Drinking water**								
Shallow tubewell is primary water source	329 (73%)	337 (76%)	309 (71%)	318 (71%)	1038 (75%)	546 (78%)	519 (74%)	504 (73%)
Has stored water at home	230 (51%)	199 (45%)	209 (48%)	229 (51%)	666 (48%)	304 (43%)	301 (43%)	331 (48%)
Reported treating water yesterday	1 (0%)	0 (0%)	0 (0%)	1 (0%)	4 (0%)	0 (0%)	0 (0%)	2 (0%)
Minutes to primary drinking water source	1 (2)	1 (6)	1 (2)	1 (2)	1 (1)	1 (5)	1 (3)	1 (2)
**Sanitation**								
Daily defecating in the open								
Adult men	19 (4%)	29 (7%)	38 (9%)	38 (9%)	97 (7%)	54 (8%)	59 (9%)	50 (7%)
Adult women	12 (3%)	16 (4%)	23 (5%)	21 (5%)	62 (4%)	29 (4%)	39 (6%)	24 (4%)
Children aged 8 to ¡15 years	9 (5%)	17 (8%)	13 (8%)	22 (11%)	53 (10%)	30 (10%)	23 (8%)	28 (10%)
Children aged 3 to ¡8 years	65 (30%)	89 (37%)	90 (40%)	92 (37%)	267 (38%)	137 (38%)	129 (39%)	134 (37%)
Children aged 0 to ¡3 years	71 (72%)	73 (75%)	68 (80%)	79 (88%)	245 (82%)	123 (79%)	128 (85%)	123 (88%)
Latrine								
Owned	271 (60%)	244 (55%)	234 (54%)	230 (51%)	750 (54%)	373 (53%)	377 (54%)	367 (53%)
Concrete slab	426 (97%)	400 (93%)	382 (93%)	399 (94%)	1251 (95%)	620 (93%)	620 (94%)	621 (94%)
Functional water seal	157 (38%)	95 (26%)	114 (32%)	111 (31%)	358 (31%)	152 (26%)	183 (31%)	155 (27%)
Visible stool on slab or floor	197 (45%)	225 (54%)	210 (52%)	222 (53%)	625 (48%)	289 (44%)	331 (51%)	298 (46%)
Owned a child potty	37 (8%)	20 (4%)	27 (6%)	21 (5%)	61 (4%)	27 (4%)	36 (5%)	30 (4%)
Human feces observed in the								
House	25 (6%)	36 (8%)	41 (9%)	36 (8%)	114 (8%)	48 (7%)	58 (8%)	49 (7%)
Child's play area	5 (1%)	4 (1%)	7 (2%)	6 (1%)	21 (2%)	7 (1%)	8 (1%)	7 (1%)
**Handwashing location**								
Within six steps of latrine								
Latrine Has water	84 (21%)	51 (13%)	38 (10%)	54 (13%)	178 (14%)	67 (10%)	62 (10%)	72 (11%)
Latrine Has soap	45 (11%)	32 (8%)	23 (6%)	27 (7%)	88 (7%)	42 (7%)	33 (5%)	36 (6%)
Within six steps of kitchen								
Kitchen Has water	48 (12%)	40 (10%)	43 (11%)	42 (10%)	118 (9%)	61 (9%)	60 (9%)	60 (9%)
Kitchen Has soap	18 (4%)	11 (3%)	19 (5%)	14 (3%)	33 (3%)	15 (2%)	22 (3%)	18 (3%)
**Nutrition**								
Household is food secure	331 (74%)	298 (67%)	308 (71%)	317 (71%)	932 (67%)	482 (69%)	479 (69%)	485 (71%)

Abbreviations: WSH, water, sanitation, and handwashing; N+WSH, nutrition plus water, sanitation, and handwashing

[a] Open defecation does not include diaper disposal of feces.

[b] Households who do not own a latrine typically share a latrine with extended family members who live in the same compound.

[c] Assessed by the Household Food Insecurity Access Scale.

Table S3: Pre-specified covariates screened for inclusion in fully adjusted models

Category	Variable
Sample	Season of collection (monsoon or dry)
	Length of time between defecation and sample placed on cold chain (ice packs/dry ice)
Child	Age (days)
	Sex
	Birth order (first born, second born or greater)
Maternal	Age (years)
	Height (cm)
	Education level (no education, primary, secondary)
Household	Number of individuals living in the compound
	Number of children <18 years in the household
	Distance in minutes to drinking water source
	Housing materials (floor, walls)
	Household assets (electricity, wardrobe, table, chair or bench, khat, chouki, working radio, black/white or color television, refrigerator, bicycle (not child's toy), motorcycle, sewing machine, mobile phone, number of cows, number of goats, number of poultry)

Table S4: **Quantity difference in $\log_{10}$ copies per gram of stool between study arms for children 14 months old.** Bold font indicates significance after correction for false discovery. Analyses employed a g-computation estimator with a two-step model including both logistic and log-linear regression using generalized linear models.

	Pathogen Quantity ($\log_{10}$ copies/g stool)[a]						Quantity Difference ($\log_{10}$ copies/g stool)[b]			
	Total		Monsoon season		Dry season		Intervention *v* Control		N+WSH *v* Intervention	
Pathogen, Arm	N[c]	Mean (SD)	N[c]	Mean (SD)	N[c]	Mean (SD)	Qty Diff (95% CI)	p[d]	Qty Diff (95% CI)	p[d]
**EAEC**										
Control	249	7.75 (1.49)	99	7.94 (1.54)	150	7.63 (1.45)	NA	NA	NA	NA
WSH	289	7.95 (1.49)	227	7.88 (1.47)	61	8.22 (1.56)	0.14 (-0.30, 0.65)	0.66	-0.21 (-0.69, 0.21)	0.37
Nutrition	251	7.62 (1.41)	178	7.58 (1.37)	73	7.73 (1.5)	-0.62 (-1.11, -0.09)	0.14	0.37 (-0.01, 0.84)	0.37
N+WSH	283	7.61 (1.51)	211	7.49 (1.5)	72	7.95 (1.52)	-0.15 (-0.57, 0.50)	0.75	NA	NA
**ST-ETEC**										
Control	58	7.21 (1.85)	30	7.08 (1.98)	28	7.34 (1.73)	NA	NA	NA	NA
WSH	81	7.53 (1.88)	75	7.51 (1.87)	6	7.77 (2.15)	-0.17 (-0.54, 0.22)	0.54	-0.16 (-0.53, 0.18)	0.37
Nutrition	63	7.71 (1.69)	56	7.68 (1.7)	7	7.94 (1.75)	-0.21 (-0.55, 0.17)	0.33	0.14 (-0.25, 0.48)	0.48
N+WSH	73	7.38 (1.7)	69	7.39 (1.72)	4	7.20 (1.68)	-0.29 (-0.67, 0.13)	0.34	NA	NA
**LT-ETEC**										
Control	61	7.03 (1.68)	21	7.64 (1.78)	40	6.70 (1.55)	NA	NA	NA	NA
WSH	68	7.26 (1.6)	61	7.17 (1.62)	7	8.01 (1.27)	-0.03 (-0.37, 0.20)	0.79	0.11 (-0.24, 0.43)	0.44
Nutrition	81	7.24 (1.78)	61	7.21 (1.75)	20	7.32 (1.89)	0.19 (-0.19, 0.54)	0.33	-0.19 (-0.59, 0.21)	0.48
N+WSH	74	7.24 (1.74)	59	7.15 (1.8)	15	7.56 (1.48)	0.00 (-0.35, 0.31)	1	NA	NA
**aEPEC**										
Control	150	6.95 (1.22)	46	7.00 (1.16)	104	6.93 (1.25)	NA	NA	NA	NA
WSH	130	6.81 (1.24)	92	6.70 (1.17)	38	7.09 (1.38)	-0.29 (-0.72, 0.13)	0.24	0.40 (0.15, 0.85)	0.08
Nutrition	141	6.78 (1.3)	95	6.78 (1.27)	46	6.77 (1.36)	-0.38 (-0.75, 0.10)	0.22	0.26 (-0.08, 0.67)	0.37
N+WSH	154	7.08 (1.3)	106	6.97 (1.22)	48	7.32 (1.43)	-0.04 (-0.39, 0.53)	0.89	NA	NA
**tEPEC**										
Control	27	7.39 (1.48)	14	7.84 (1.14)	13	6.90 (1.69)	NA	NA	NA	NA
WSH	54	7.45 (1.39)	47	7.53 (1.37)	7	6.92 (1.54)	0.08 (-0.31, 0.44)	0.67	-0.28 (-0.56, -0.03)	0.08
Nutrition	47	7.14 (1.44)	44	7.25 (1.43)	3	5.59 (0.43)	0.03 (-0.25, 0.30)	0.77	-0.24 (-0.53, 0.05)	0.37
N+WSH	36	7.13 (1.13)	35	7.12 (1.15)	1	7.32 (NA)	-0.04 (-0.31, 0.25)	0.82	NA	NA
**STEC**										
Control	36	5.60 (0.7)	22	5.64 (0.77)	14	5.54 (0.59)	NA	NA	NA	NA
WSH	47	5.83 (0.82)	39	5.82 (0.8)	8	5.90 (0.94)	0.05 (-0.18, 0.22)	0.67	-0.09 (-0.29, 0.07)	0.37
Nutrition	41	5.84 (0.86)	33	5.81 (0.82)	8	5.98 (1.04)	-0.03 (-0.24, 0.16)	0.75	-0.08 (-0.32, 0.08)	0.48
N+WSH	36	5.84 (0.68)	28	5.87 (0.71)	8	5.76 (0.62)	-0.05 (-0.29, 0.14)	0.75	NA	NA
Aeromonas										
Control	21	5.85 (0.67)	4	6.42 (0.45)	17	5.71 (0.65)	NA	NA	NA	NA
WSH	19	5.87 (0.67)	19	5.87 (0.67)	0	NA	-0.06 (-0.19, 0.10)	0.66	0.15 (-0.05, 0.30)	0.28
Nutrition	21	6.04 (0.84)	18	6.16 (0.85)	3	5.33 (0.26)	-0.04 (-0.17, 0.14)	0.71	0.03 (-0.16, 0.19)	0.78
N+WSH	24	6.14 (0.81)	23	6.18 (0.79)	1	5.04 (NA)	-0.02 (-0.20, 0.16)	0.88	NA	NA
B. fragilis										
Control	61	8.28 (1.27)	17	8.37 (1.27)	44	8.24 (1.28)	NA	NA	NA	NA
WSH	85	8.32 (1.36)	66	8.17 (1.34)	18	8.76 (1.36)	0.24 (-0.13, 0.60)	0.24	0.22 (-0.27, 0.62)	0.37
Nutrition	79	8.05 (1.51)	55	7.85 (1.5)	24	8.52 (1.46)	0.20 (-0.24, 0.60)	0.44	0.33 (-0.09, 0.83)	0.37
N+WSH	98	8.10 (1.47)	74	8.08 (1.49)	24	8.19 (1.44)	0.43 (-0.01, 0.83)	0.2	NA	NA
Campylobacter spp.										
Control	136	7.32 (1.42)	48	7.47 (1.59)	88	7.24 (1.32)	NA	NA	NA	NA
WSH	178	7.54 (1.4)	146	7.59 (1.32)	32	7.28 (1.71)	0.24 (-0.41, 0.82)	0.59	-0.19 (-0.66, 0.21)	0.37
Nutrition	181	7.27 (1.39)	133	7.38 (1.35)	48	6.96 (1.47)	0.06 (-0.44, 0.51)	0.75	-0.28 (-0.73, 0.16)	0.4
N+WSH	166	7.46 (1.54)	134	7.44 (1.5)	32	7.54 (1.74)	-0.09 (-0.59, 0.38)	0.79	NA	NA

(continued)

	Pathogen Quantity ($\log_{10}$ copies/g stool)[a]						Quantity Difference ($\log_{10}$ copies/g stool)[b]			
	Total		Monsoon season		Dry season		Intervention *v* Control		N+WSH *v* Intervention	
Pathogen, Arm	N[c]	Mean (SD)	N[c]	Mean (SD)	N[c]	Mean (SD)	Qty Diff (95% CI)	p[d]	Qty Diff (95% CI)	p[d]
C. difficile										
Control	27	6.69 (1.48)	10	6.46 (1.54)	17	6.82 (1.47)	NA	NA	NA	NA
WSH	32	6.69 (1.15)	26	6.56 (1.15)	6	7.26 (1.06)	-0.03 (-0.25, 0.22)	0.79	0.13 (-0.14, 0.34)	0.37
Nutrition	33	7.01 (1.14)	20	6.75 (1.16)	13	7.41 (1.04)	0.07 (-0.20, 0.32)	0.65	0.06 (-0.14, 0.33)	0.67
N+WSH	39	6.88 (1.36)	26	7.20 (1.08)	13	6.23 (1.66)	0.09 (-0.14, 0.36)	0.75	NA	NA
Plesiomonas										
Control	45	6.28 (0.98)	12	6.54 (0.71)	33	6.18 (1.06)	NA	NA	NA	NA
WSH	53	6.41 (0.94)	50	6.47 (0.92)	3	5.41 (0.52)	-0.07 (-0.31, 0.21)	0.66	0.20 (-0.08, 0.45)	0.26
Nutrition	54	6.39 (0.93)	45	6.33 (0.96)	9	6.72 (0.72)	0.05 (-0.19, 0.32)	0.75	0.13 (-0.22, 0.45)	0.48
N+WSH	65	6.65 (1.14)	51	6.55 (1.04)	14	7.03 (1.42)	0.14 (-0.13, 0.41)	0.47	NA	NA
Shigella spp./EIEC										
Control	41	7.32 (1.37)	19	7.23 (1.51)	22	7.39 (1.27)	NA	NA	NA	NA
WSH	48	7.22 (1.45)	45	7.32 (1.45)	3	5.79 (0.48)	-0.30 (-0.63, 0.06)	0.13	0.13 (-0.13, 0.53)	0.43
Nutrition	59	7.65 (1.2)	46	7.68 (1.18)	13	7.55 (1.3)	0.12 (-0.22, 0.55)	0.65	-0.18 (-0.58, 0.15)	0.48
N+WSH	54	7.63 (1.47)	49	7.58 (1.49)	5	8.03 (1.28)	-0.09 (-0.38, 0.28)	0.75	NA	NA
**Adenovirus 40/41**										
Control	34	7.86 (2.72)	27	7.72 (2.74)	7	8.38 (2.8)	NA	NA	NA	NA
WSH	21	7.49 (2.06)	18	7.62 (2.18)	3	6.67 (0.81)	-0.47 (-0.84, -0.10)	0.05	0.04 (-0.19, 0.28)	0.65
Nutrition	29	7.28 (2.29)	20	7.05 (2.42)	9	7.78 (2.04)	-0.25 (-0.65, 0.16)	0.33	-0.03 (-0.35, 0.23)	0.84
N+WSH	25	7.09 (2.35)	17	7.08 (2.23)	8	7.12 (2.76)	-0.30 (-0.68, 0.12)	0.34	NA	NA
**Norovirus**										
Control	75	5.61 (1.29)	26	5.7 (1.51)	49	5.56 (1.17)	NA	NA	NA	NA
WSH	42	5.32 (1.12)	35	5.15 (1.06)	7	6.17 (1.1)	**-0.45 (-0.70, -0.21)**	**0.02**	0.28 (0.05, 0.50)	0.08
Nutrition	62	5.44 (1.35)	33	5.23 (1.41)	29	5.67 (1.26)	-0.16 (-0.41, 0.17)	0.33	0.06 (-0.24, 0.28)	0.67
N+WSH	64	5.56 (1.28)	46	5.71 (1.3)	18	5.17 (1.18)	-0.19 (-0.46, 0.10)	0.34	NA	NA
**Sapovirus**										
Control	61	7.18 (1.49)	18	7.04 (1.4)	43	7.23 (1.53)	NA	NA	NA	NA
WSH	32	7.27 (1.8)	22	6.94 (1.57)	9	7.80 (2.17)	**-0.38 (-0.74, -0.07)**	**0.04**	0.16 (-0.13, 0.45)	0.37
Nutrition	36	7.05 (1.37)	22	6.97 (1.26)	14	7.16 (1.56)	-0.33 (-0.72, -0.03)	0.16	0.09 (-0.21, 0.39)	0.65
N+WSH	42	7.33 (1.57)	26	7.33 (1.62)	16	7.34 (1.53)	-0.18 (-0.53, 0.14)	0.49	NA	NA
Cryptosporidium										
Control	26	6.79 (1.44)	11	6.85 (1.27)	15	6.76 (1.59)	NA	NA	NA	NA
WSH	53	6.69 (1.48)	47	6.76 (1.53)	6	6.12 (0.8)	0.08 (-0.27, 0.33)	0.66	-0.06 (-0.28, 0.25)	0.62
Nutrition	43	7.00 (1.87)	33	7.01 (1.78)	10	6.98 (2.24)	0.18 (-0.18, 0.36)	0.33	-0.01 (-0.29, 0.30)	0.93
N+WSH	49	6.84 (1.38)	36	6.91 (1.47)	13	6.68 (1.16)	0.18 (-0.04, 0.46)	0.34	NA	NA
E. bieneusi										
Control	35	6.64 (1.34)	11	5.80 (0.95)	24	7.03 (1.33)	NA	NA	NA	NA
WSH	36	6.01 (1.33)	29	6.16 (1.4)	7	5.39 (0.76)	-0.15 (-0.39, 0.06)	0.24	0.05 (-0.17, 0.25)	0.6
Nutrition	41	6.53 (1.47)	32	6.54 (1.56)	9	6.51 (1.15)	-0.14 (-0.37, 0.14)	0.33	-0.05 (-0.31, 0.12)	0.67
N+WSH	32	7.01 (1.48)	26	6.83 (1.42)	6	7.78 (1.65)	-0.06 (-0.28, 0.14)	0.75	NA	NA
Giardia										
Control	45	8.61 (2.06)	9	7.74 (1.48)	36	8.82 (2.14)	NA	NA	NA	NA
WSH	53	8.81 (1.85)	42	8.81 (1.89)	11	8.82 (1.8)	-0.08 (-0.49, 0.30)	0.67	-0.16 (-0.49, 0.22)	0.37
Nutrition	48	8.71 (1.92)	29	9.09 (1.54)	19	8.12 (2.31)	0.08 (-0.26, 0.57)	0.75	-0.16 (-0.52, 0.19)	0.48
N+WSH	45	8.49 (1.76)	34	8.70 (1.68)	11	7.86 (1.92)	-0.12 (-0.53, 0.27)	0.75	NA	NA

CI, confidence interval; NA, not applicable; WSH, water, sanitation, and handwashing; N+WSH, nutrition plus water, sanitation, and handwashing

[a] Mean quantity measured in $\log_{10}$ copies per gram of stool among positive stools only.

[b] Quantity difference includes positive and negative stools and is adjusted for pre-specified covariates with a likelihood ratio test $p < 0.1$ in bivariate analysis with the outcome: household food insecurity; child age, sex, and birth order; season of sample collection; time until sample placed on cold chain; mother's age, height, and education level; number of children < 18 years in the household; number of individuals living in the compound; distance in minutes to the primary water source; household floor and wall materials; household assets.

[c] Number of positive stools over which the mean was calculated

[d] p values shown are adjusted for false discovery rate using the Benjamini-Hochberg procedure.

Table S5: **Unadjusted, age/season adjusted, and fully adjusted (with only non-diarrheal stools) difference in $\log_{10}$ copies per gram of stool between study arms for children 14 months old.** Bold font indicates significance after correction for false discovery. Analyses employed a g-computation estimator with a two-step model including both logistic and log-linear regression using generalized linear models.

	Unadjusted Model				Age & Season Adjusted Model				Fully Adjusted Model w/ non-diarrheal samples[a]			
	Intervention v Control		N+WSH v Intervention		Intervention v Control		N+WSH v Intervention		Intervention v Control		N+WSH v Intervention	
Pathogen, Arm	Qty Diff (95% CI)	p[b]	Qty Diff (95% CI)	p[b]	Qty Diff (95% CI)	p[b]	Qty Diff (95% CI)	p[b]	Qty Diff (95% CI)	p[b]	Qty Diff (95% CI)	p[b]
**EAEC**												
WSH	0.36 (-0.09, 0.80)	0.08	-0.21 (-0.66, 0.20)	0.37	0.04 (-0.46, 0.53)	0.89	-0.22 (-0.66, 0.20)	0.32	0.28 (-0.19, 0.90)	0.4	-0.34 (-0.74, 0.08)	0.14
Nutrition	-0.36 (-0.82, 0.13)	0.14	0.45 (0.02, 0.87)	0.14	**-0.67 (-1.16, -0.16)**	**0.04**	0.44 (-0.02, 0.84)	0.22	-0.47 (-0.93, 0.10)	0.14	0.40 (0.00, 0.86)	0.47
N+WSH	0.10 (-0.36, 0.63)	0.76	NA	NA	-0.11 (-0.72, 0.46)	0.89	NA	NA	-0.11 (-0.49, 0.58)	0.71	NA	NA
**ST-ETEC**												
WSH	0.31 (-0.09, 0.73)	0.11	-0.14 (-0.53, 0.27)	0.5	-0.06 (-0.49, 0.37)	0.79	-0.09 (-0.45, 0.26)	0.6	-0.30 (-0.69, 0.17)	0.21	-0.02 (-0.38, 0.32)	0.88
Nutrition	0.10 (-0.30, 0.51)	0.57	0.08 (-0.31, 0.47)	0.63	-0.21 (-0.58, 0.15)	0.38	0.02 (-0.36, 0.39)	0.88	-0.32 (-0.64, 0.25)	0.32	0.20 (-0.17, 0.59)	0.56
N+WSH	0.19 (-0.18, 0.54)	0.29	NA	NA	-0.23 (-0.64, 0.15)	0.33	NA	NA	-0.33 (-0.78, 0.13)	0.27	NA	NA
**LT-ETEC**												
WSH	0.04 (-0.28, 0.36)	0.79	0.14 (-0.25, 0.47)	0.43	-0.20 (-0.54, 0.12)	0.24	0.12 (-0.22, 0.47)	0.49	-0.11 (-0.49, 0.19)	0.52	0.11 (-0.25, 0.45)	0.51
Nutrition	0.30 (-0.07, 0.63)	0.11	-0.15 (-0.53, 0.29)	0.63	0.20 (-0.24, 0.55)	0.38	-0.13 (-0.55, 0.23)	0.54	0.22 (-0.25, 0.56)	0.33	-0.27 (-0.66, 0.16)	0.56
N+WSH	0.15 (-0.19, 0.49)	0.39	NA	NA	0.03 (-0.35, 0.48)	0.94	NA	NA	-0.02 (-0.42, 0.33)	0.88	NA	NA
**aEPEC**												
WSH	**-0.60 (-0.97, -0.15)**	**0.01**	**0.50 (0.16, 0.88)**	**0.02**	-0.44 (-0.90, 0.03)	0.09	**0.50 (0.13, 0.86)**	**0.01**	-0.33 (-0.78, 0.13)	0.21	0.29 (-0.04, 0.82)	0.29
Nutrition	-0.34 (-0.76, 0.06)	0.11	0.29 (-0.08, 0.68)	0.46	-0.38 (-0.82, 0.09)	0.21	0.28 (-0.08, 0.66)	0.38	-0.35 (-0.77, 0.10)	0.18	0.13 (-0.21, 0.63)	0.71
N+WSH	-0.07 (-0.43, 0.35)	0.76	NA	NA	-0.02 (-0.47, 0.48)	0.94	NA	NA	-0.31 (-0.75, 0.29)	0.33	NA	NA
**tEPEC**												
WSH	**0.36 (0.10, 0.66)**	**0.01**	-0.31 (-0.56, -0.03)	0.06	0.17 (-0.16, 0.48)	0.27	-0.28 (-0.53, -0.02)	0.08	0.17 (-0.23, 0.56)	0.48	**-0.36 (-0.66, -0.08)**	**0.04**
Nutrition	0.26 (-0.02, 0.57)	0.11	-0.17 (-0.48, 0.12)	0.63	0.02 (-0.26, 0.32)	0.88	-0.23 (-0.51, 0.05)	0.38	-0.05 (-0.34, 0.25)	0.74	-0.09 (-0.43, 0.16)	0.71
N+WSH	0.09 (-0.13, 0.32)	0.49	NA	NA	-0.05 (-0.32, 0.23)	0.89	NA	NA	-0.22 (-0.49, 0.06)	0.27	NA	NA
**STEC**												
WSH	0.10 (-0.08, 0.28)	0.25	-0.12 (-0.29, 0.07)	0.35	0.09 (-0.12, 0.28)	0.36	-0.10 (-0.30, 0.07)	0.29	0.02 (-0.21, 0.25)	0.86	-0.06 (-0.27, 0.15)	0.51
Nutrition	0.06 (-0.14, 0.24)	0.56	-0.06 (-0.27, 0.13)	0.63	0.01 (-0.20, 0.22)	0.88	-0.08 (-0.28, 0.11)	0.54	-0.02 (-0.29, 0.20)	0.87	-0.05 (-0.28, 0.18)	0.71
N+WSH	-0.01 (-0.19, 0.17)	0.89	NA	NA	-0.03 (-0.23, 0.17)	0.9	NA	NA	-0.02 (-0.28, 0.22)	0.88	NA	NA
Aeromonas												
WSH	-0.06 (-0.19, 0.11)	0.5	0.09 (-0.09, 0.26)	0.37	-0.13 (-0.28, 0.01)	0.09	0.10 (-0.05, 0.28)	0.29	-0.13 (-0.25, 0.05)	0.14	0.12 (-0.05, 0.27)	0.17
Nutrition	-0.01 (-0.16, 0.17)	0.87	0.04 (-0.15, 0.22)	0.63	-0.07 (-0.21, 0.09)	0.38	0.02 (-0.17, 0.20)	0.83	-0.08 (-0.23, 0.09)	0.33	0.03 (-0.18, 0.20)	0.71
N+WSH	0.03 (-0.13, 0.21)	0.76	NA	NA	-0.07 (-0.22, 0.11)	0.48	NA	NA	-0.04 (-0.21, 0.14)	0.68	NA	NA
B. fragilis												
WSH	0.31 (-0.07, 0.70)	0.08	0.21 (-0.25, 0.65)	0.37	0.45 (0.01, 0.93)	0.07	0.26 (-0.21, 0.67)	0.29	0.08 (-0.32, 0.55)	0.75	0.35 (-0.12, 0.81)	0.17
Nutrition	0.18 (-0.22, 0.63)	0.45	0.33 (-0.13, 0.81)	0.5	0.36 (-0.06, 0.83)	0.21	0.31 (-0.13, 0.82)	0.46	0.23 (-0.24, 0.71)	0.34	0.26 (-0.21, 0.84)	0.56
N+WSH	**0.54 (0.11, 0.93)**	**0.03**	NA	NA	0.53 (0.08, 1.00)	0.13	NA	NA	0.41 (-0.02, 0.91)	0.27	NA	NA
Campylobacter spp.												
WSH	0.51 (0.01, 1.05)	0.05	-0.14 (-0.62, 0.31)	0.53	0.20 (-0.35, 0.85)	0.46	-0.12 (-0.57, 0.30)	0.56	0.29 (-0.38, 0.90)	0.4	-0.24 (-0.71, 0.24)	0.33
Nutrition	0.54 (0.05, 0.99)	0.07	-0.18 (-0.67, 0.32)	0.63	0.12 (-0.35, 0.59)	0.59	-0.25 (-0.70, 0.19)	0.53	0.15 (-0.39, 0.66)	0.63	-0.31 (-0.77, 0.24)	0.56
N+WSH	0.37 (-0.12, 0.86)	0.17	NA	NA	-0.10 (-0.57, 0.41)	0.89	NA	NA	-0.15 (-0.65, 0.48)	0.68	NA	NA

(continued)

	Unadjusted Model				Age & Season Adjusted Model				Fully Adjusted Model w/ non-diarrheal samples[a]			
	Intervention v Control		N+WSH v Intervention		Intervention v Control		N+WSH v Intervention		Intervention v Control		N+WSH v Intervention	
Pathogen, Arm	Qty Diff (95% CI)	p[b]	Qty Diff (95% CI)	p[b]	Qty Diff (95% CI)	p[b]	Qty Diff (95% CI)	p[b]	Qty Diff (95% CI)	p[b]	Qty Diff (95% CI)	p[b]
C. difficile												
WSH	0.03 (-0.22, 0.26)	0.79	0.12 (-0.13, 0.36)	0.37	-0.01 (-0.30, 0.25)	0.93	0.14 (-0.12, 0.37)	0.29	0.01 (-0.20, 0.26)	0.9	0.09 (-0.14, 0.35)	0.5
Nutrition	0.09 (-0.16, 0.34)	0.45	0.07 (-0.16, 0.31)	0.63	0.09 (-0.16, 0.34)	0.44	0.08 (-0.16, 0.33)	0.54	0.13 (-0.11, 0.35)	0.33	0.12 (-0.09, 0.39)	0.56
N+WSH	0.16 (-0.09, 0.41)	0.27	NA	NA	0.17 (-0.11, 0.46)	0.33	NA	NA	0.09 (-0.16, 0.36)	0.57	NA	NA
Plesiomonas												
WSH	0.06 (-0.21, 0.34)	0.68	0.23 (-0.06, 0.48)	0.12	-0.17 (-0.43, 0.09)	0.23	0.22 (-0.04, 0.49)	0.13	-0.03 (-0.30, 0.25)	0.86	0.24 (-0.02, 0.60)	0.17
Nutrition	0.09 (-0.18, 0.41)	0.56	0.17 (-0.15, 0.51)	0.63	0.04 (-0.23, 0.35)	0.79	0.14 (-0.19, 0.47)	0.54	0.07 (-0.22, 0.40)	0.7	0.16 (-0.19, 0.50)	0.56
N+WSH	0.28 (0.01, 0.57)	0.05	NA	NA	0.17 (-0.11, 0.51)	0.33	NA	NA	0.16 (-0.06, 0.49)	0.33	NA	NA
Shigella spp./EIEC												
WSH	0.02 (-0.31, 0.37)	0.87	0.16 (-0.17, 0.51)	0.37	-0.16 (-0.47, 0.15)	0.27	0.19 (-0.15, 0.51)	0.29	-0.34 (-0.76, 0.05)	0.21	0.20 (-0.11, 0.57)	0.29
Nutrition	0.29 (-0.06, 0.69)	0.11	-0.09 (-0.50, 0.29)	0.63	0.21 (-0.18, 0.62)	0.38	-0.13 (-0.53, 0.24)	0.54	0.02 (-0.31, 0.42)	0.88	0.00 (-0.40, 0.38)	1
N+WSH	0.20 (-0.14, 0.52)	0.27	NA	NA	0.06 (-0.30, 0.39)	0.89	NA	NA	-0.08 (-0.42, 0.29)	0.69	NA	NA
**Adenovirus 40/41**												
WSH	-0.30 (-0.62, 0.03)	0.08	0.03 (-0.20, 0.28)	0.79	-0.51 (-0.96, -0.13)	0.06	0.04 (-0.19, 0.29)	0.69	-0.45 (-0.96, -0.02)	0.21	0.02 (-0.17, 0.25)	0.83
Nutrition	-0.17 (-0.58, 0.16)	0.45	-0.09 (-0.36, 0.20)	0.63	-0.22 (-0.69, 0.18)	0.38	-0.08 (-0.37, 0.21)	0.61	-0.13 (-0.61, 0.31)	0.63	-0.10 (-0.40, 0.21)	0.71
N+WSH	-0.24 (-0.60, 0.09)	0.27	NA	NA	-0.39 (-0.83, 0.03)	0.32	NA	NA	-0.28 (-0.70, 0.09)	0.28	NA	NA
**Norovirus**												
WSH	**-0.45 (-0.69, -0.21)**	**0.01**	**0.26 (0.05, 0.49)**	**0.02**	**-0.52 (-0.85, -0.20)**	**0.03**	**0.27 (0.04, 0.48)**	**0.01**	**-0.40 (-0.65, -0.17)**	**0.02**	0.22 (-0.02, 0.47)	0.14
Nutrition	-0.23 (-0.50, 0.06)	0.11	0.03 (-0.24, 0.28)	0.79	-0.19 (-0.49, 0.13)	0.38	0.05 (-0.22, 0.29)	0.76	-0.17 (-0.43, 0.18)	0.33	0.05 (-0.22, 0.27)	0.71
N+WSH	-0.22 (-0.48, 0.07)	0.17	NA	NA	-0.20 (-0.56, 0.10)	0.33	NA	NA	-0.23 (-0.48, 0.05)	0.27	NA	NA
**Sapovirus**												
WSH	**-0.56 (-0.89, -0.25)**	**0.01**	0.18 (-0.13, 0.46)	0.37	**-0.46 (-0.83, -0.11)**	**0.02**	0.19 (-0.10, 0.46)	0.29	-0.26 (-0.70, 0.01)	0.27	0.27 (-0.04, 0.53)	0.14
Nutrition	**-0.49 (-0.83, -0.16)**	**0.02**	0.12 (-0.19, 0.40)	0.63	-0.41 (-0.74, -0.08)	0.05	0.13 (-0.17, 0.42)	0.54	-0.31 (-0.67, -0.02)	0.14	0.18 (-0.11, 0.48)	0.56
N+WSH	**-0.39 (-0.70, -0.06)**	**0.04**	NA	NA	-0.24 (-0.54, 0.12)	0.32	NA	NA	-0.10 (-0.48, 0.21)	0.65	NA	NA
Cryptosporidium												
WSH	**0.32 (0.08, 0.55)**	**0.01**	-0.01 (-0.29, 0.27)	0.91	0.06 (-0.22, 0.34)	0.69	0.00 (-0.27, 0.30)	1	0.14 (-0.23, 0.46)	0.49	-0.08 (-0.32, 0.23)	0.51
Nutrition	0.24 (-0.01, 0.50)	0.1	0.06 (-0.23, 0.31)	0.63	0.11 (-0.16, 0.37)	0.44	0.04 (-0.26, 0.31)	0.83	0.26 (-0.06, 0.47)	0.14	-0.06 (-0.30, 0.28)	0.71
N+WSH	**0.30 (0.06, 0.54)**	**0.03**	NA	NA	0.21 (-0.08, 0.47)	0.32	NA	NA	0.20 (-0.06, 0.49)	0.27	NA	NA
E. bieneusi												
WSH	-0.10 (-0.32, 0.12)	0.38	0.05 (-0.16, 0.25)	0.58	-0.13 (-0.38, 0.07)	0.26	0.06 (-0.15, 0.26)	0.56	-0.15 (-0.43, 0.07)	0.35	0.08 (-0.16, 0.28)	0.48
Nutrition	0.04 (-0.22, 0.31)	0.79	-0.09 (-0.32, 0.14)	0.63	-0.07 (-0.32, 0.22)	0.59	-0.09 (-0.34, 0.12)	0.54	-0.16 (-0.42, 0.06)	0.24	-0.04 (-0.29, 0.17)	0.71
N+WSH	-0.03 (-0.27, 0.18)	0.76	NA	NA	-0.01 (-0.27, 0.23)	0.94	NA	NA	-0.09 (-0.37, 0.11)	0.57	NA	NA
Giardia												
WSH	0.11 (-0.34, 0.48)	0.62	-0.15 (-0.53, 0.20)	0.41	0.26 (-0.20, 0.62)	0.23	-0.16 (-0.54, 0.18)	0.39	-0.15 (-0.54, 0.33)	0.52	-0.14 (-0.47, 0.21)	0.48
Nutrition	0.03 (-0.35, 0.44)	0.87	-0.10 (-0.47, 0.23)	0.63	0.18 (-0.21, 0.68)	0.44	-0.12 (-0.47, 0.22)	0.54	-0.12 (-0.53, 0.32)	0.66	0.06 (-0.29, 0.41)	0.71
N+WSH	-0.07 (-0.46, 0.25)	0.76	NA	NA	0.01 (-0.36, 0.34)	0.94	NA	NA	-0.26 (-0.67, 0.18)	0.33	NA	NA

CI, confidence interval; NA, not applicable; WSH, water, sanitation, and handwashing; N+WSH, nutrition plus water, sanitation, and handwashing

[a] Non-diarrheal samples include those from children with no reported diarrheal symptoms in the previous seven days.

[b] p values shown are adjusted for false discovery rate using the Benjamini-Hochberg procedure.

Table S6: **Prevalence difference (PD) of individual pathogens between arms in children at age 14 months.** Bold font indicates significance after correction for false discovery rate using the Benjamini-Hochberg procedure. Unadjusted and adjusted analyses used a generalized linear model. IPCW adjusted analysis corrected for potential bias due to informative censoring using targeted maximum likelihood estimation with data-adaptive model selection.

	Unadjusted Model				Age & Season Adjusted Model				Fully Adjusted Model w/ non-diarrheal samples[a]				IPCW Adjusted Model[b]			
**Pathogen**	Intervention v Control		N+WSH v Intervention		Intervention v Control		N+WSH v Intervention		Intervention v Control		N+WSH v Intervention		Intervention v Control		N+WSH v Intervention	
Arm	PD (95% CI)	p[c]	PD (95% CI)	p[c]	PD (95% CI)	p[c]	PD (95% CI)	p[c]	PD (95% CI)	p[c]	PD (95% CI)	p[c]	PD (95% CI)	p[c]	PD (95% CI)	p[c]
**EAEC**																
WSH	0.03 (-0.03, 0.1)	0.55	0.01 (-0.06, 0.07)	0.86	-0.02 (-0.09, 0.06)	0.74	0.01 (-0.05, 0.07)	0.80	0.01 (-0.07, 0.09)	0.89	0 (-0.06, 0.06)	0.95	0.01 (-0.05, 0.07)	0.82	0.01 (-0.06, 0.07)	0.86
Nutrition	-0.04 (-0.11, 0.03)	0.43	0.08 (0.02, 0.15)	0.22	-0.09 (-0.17, -0.02)	0.15	0.07 (0.01, 0.14)	0.34	-0.07 (-0.16, 0.01)	0.57	0.07 (0, 0.13)	0.71	**-0.09 (-0.15, -0.03)**	**0.03**	0.08 (0.02, 0.14)	0.12
N+WSH	0.04 (-0.03, 0.11)	0.52	NA	NA	0 (-0.08, 0.08)	1.00	NA	NA	0.01 (-0.07, 0.1)	0.78	NA	NA	0.02 (-0.05, 0.08)	0.73	NA	NA
**ST-ETEC**																
WSH	0.05 (-0.02, 0.11)	0.37	-0.02 (-0.08, 0.05)	0.76	-0.02 (-0.09, 0.05)	0.72	-0.01 (-0.07, 0.05)	0.80	-0.04 (-0.11, 0.03)	0.57	0.01 (-0.05, 0.06)	0.88	-0.03 (-0.1, 0.03)	0.69	-0.01 (-0.07, 0.05)	0.86
Nutrition	0 (-0.07, 0.07)	0.98	0.03 (-0.04, 0.1)	0.76	-0.05 (-0.11, 0.02)	0.60	0.02 (-0.05, 0.08)	0.79	-0.06 (-0.13, 0.01)	0.57	0.04 (-0.02, 0.11)	0.71	-0.04 (-0.11, 0.03)	0.63	0.01 (-0.06, 0.07)	0.93
N+WSH	0.03 (-0.03, 0.09)	0.53	NA	NA	-0.04 (-0.11, 0.02)	0.59	NA	NA	-0.04 (-0.11, 0.03)	0.66	NA	NA	-0.04 (-0.09, 0.01)	0.31	NA	NA
**LT-ETEC**																
WSH	0 (-0.06, 0.06)	0.99	0.02 (-0.04, 0.09)	0.76	-0.04 (-0.1, 0.02)	0.40	0.02 (-0.04, 0.09)	0.66	-0.06 (-0.12, -0.01)	0.17	0.02 (-0.04, 0.09)	0.64	-0.02 (-0.08, 0.03)	0.69	0.01 (-0.05, 0.08)	0.86
Nutrition	0.04 (-0.02, 0.11)	0.41	-0.02 (-0.09, 0.05)	0.76	0.03 (-0.04, 0.1)	0.66	-0.03 (-0.1, 0.04)	0.78	0.02 (-0.05, 0.09)	0.76	-0.03 (-0.1, 0.04)	0.71	0.02 (-0.04, 0.09)	0.75	-0.03 (-0.1, 0.04)	0.81
N+WSH	0.02 (-0.04, 0.08)	0.60	NA	NA	0.01 (-0.06, 0.07)	1.00	NA	NA	-0.01 (-0.07, 0.05)	0.78	NA	NA	0 (-0.06, 0.05)	0.90	NA	NA
**aEPEC**																
WSH	**-0.1 (-0.18, -0.03)**	**0.03**	0.08 (0.01, 0.15)	0.22	-0.08 (-0.16, 0)	0.28	0.08 (0.01, 0.15)	0.23	-0.05 (-0.14, 0.03)	0.47	0.06 (-0.02, 0.13)	0.41	-0.07 (-0.14, 0)	0.39	0.07 (0, 0.14)	0.46
Nutrition	-0.06 (-0.13, 0.02)	0.41	0.03 (-0.04, 0.1)	0.76	-0.06 (-0.14, 0.02)	0.60	0.03 (-0.04, 0.1)	0.78	-0.05 (-0.13, 0.03)	0.64	0.02 (-0.06, 0.1)	0.89	-0.06 (-0.12, 0.01)	0.38	0.03 (-0.04, 0.1)	0.81
N+WSH	-0.02 (-0.1, 0.05)	0.67	NA	NA	-0.02 (-0.1, 0.07)	0.98	NA	NA	-0.03 (-0.13, 0.07)	0.67	NA	NA	-0.01 (-0.09, 0.07)	0.85	NA	NA
**tEPEC**																
WSH	**0.07 (0.02, 0.11)**	**0.03**	-0.05 (-0.1, 0)	0.40	0.04 (-0.01, 0.09)	0.38	-0.04 (-0.09, 0)	0.40	0.04 (-0.02, 0.09)	0.47	-0.05 (-0.11, 0)	0.37	0.02 (-0.03, 0.08)	0.69	-0.04 (-0.09, 0.01)	0.60
Nutrition	0.05 (0, 0.1)	0.24	-0.03 (-0.09, 0.02)	0.76	0.02 (-0.03, 0.07)	0.66	-0.04 (-0.09, 0.01)	0.70	0.01 (-0.05, 0.06)	0.88	-0.02 (-0.07, 0.03)	0.71	0.01 (-0.04, 0.06)	0.75	-0.04 (-0.08, 0.01)	0.70
N+WSH	0.02 (-0.02, 0.06)	0.53	NA	NA	0 (-0.04, 0.04)	1.00	NA	NA	-0.02 (-0.06, 0.03)	0.66	NA	NA	-0.02 (-0.05, 0.01)	0.46	NA	NA
**STEC**																
WSH	0.02 (-0.03, 0.07)	0.74	-0.03 (-0.07, 0.02)	0.63	0 (-0.05, 0.06)	0.88	-0.03 (-0.07, 0.02)	0.61	-0.01 (-0.07, 0.05)	0.88	-0.02 (-0.08, 0.03)	0.64	-0.03 (-0.07, 0.01)	0.63	-0.03 (-0.07, 0.02)	0.70
Nutrition	0.01 (-0.04, 0.06)	0.93	-0.02 (-0.06, 0.03)	0.76	-0.01 (-0.06, 0.04)	0.76	-0.02 (-0.06, 0.03)	0.78	-0.02 (-0.08, 0.04)	0.76	-0.02 (-0.07, 0.04)	0.85	-0.02 (-0.07, 0.02)	0.63	-0.03 (-0.07, 0.02)	0.81
N+WSH	-0.01 (-0.06, 0.04)	0.79	NA	NA	-0.02 (-0.07, 0.03)	0.86	NA	NA	-0.02 (-0.09, 0.04)	0.66	NA	NA	-0.04 (-0.08, 0)	0.22	NA	NA
Aeromonas																
WSH	-0.01 (-0.05, 0.02)	0.78	0.02 (-0.02, 0.06)	0.76	-0.02 (-0.06, 0.01)	0.36	0.02 (-0.02, 0.06)	0.66	-0.03 (-0.06, 0.01)	0.47	0.02 (-0.01, 0.06)	0.57	-0.01 (-0.05, 0.02)	0.69	0.02 (-0.01, 0.06)	0.70
Nutrition	0 (-0.04, 0.03)	0.93	0.01 (-0.03, 0.05)	0.76	-0.01 (-0.05, 0.02)	0.70	0 (-0.04, 0.04)	0.89	-0.01 (-0.05, 0.03)	0.76	0.01 (-0.03, 0.05)	0.89	-0.01 (-0.05, 0.03)	0.76	0 (-0.03, 0.04)	0.93
N+WSH	0 (-0.04, 0.04)	0.86	NA	NA	-0.01 (-0.05, 0.03)	0.96	NA	NA	-0.01 (-0.05, 0.04)	0.78	NA	NA	0.01 (-0.03, 0.05)	0.82	NA	NA
B. fragilis																
WSH	0.05 (-0.01, 0.11)	0.27	0.04 (-0.03, 0.11)	0.63	0.06 (-0.01, 0.13)	0.32	0.05 (-0.02, 0.12)	0.61	0.02 (-0.04, 0.09)	0.61	0.06 (-0.01, 0.13)	0.37	0.04 (-0.02, 0.1)	0.58	0.04 (-0.03, 0.11)	0.70
Nutrition	0.04 (-0.02, 0.1)	0.43	0.05 (-0.02, 0.12)	0.76	0.06 (0, 0.13)	0.40	0.05 (-0.02, 0.12)	0.70	0.04 (-0.03, 0.12)	0.64	0.05 (-0.03, 0.13)	0.71	0.04 (-0.02, 0.11)	0.63	0.04 (-0.03, 0.12)	0.81
N+WSH	0.09 (0.02, 0.16)	0.13	NA	NA	0.08 (0.01, 0.16)	0.36	NA	NA	0.09 (0.01, 0.16)	0.39	NA	NA	0.08 (0.02, 0.15)	0.10	NA	NA
Campylobacter spp.																
WSH	0.07 (-0.01, 0.16)	0.22	-0.02 (-0.1, 0.06)	0.76	0.03 (-0.06, 0.12)	0.72	-0.02 (-0.09, 0.06)	0.80	0.04 (-0.07, 0.14)	0.61	-0.03 (-0.11, 0.05)	0.64	0.02 (-0.06, 0.1)	0.80	-0.01 (-0.09, 0.06)	0.86
Nutrition	0.1 (0.02, 0.19)	0.14	-0.05 (-0.13, 0.03)	0.76	0.05 (-0.03, 0.13)	0.66	-0.06 (-0.13, 0.01)	0.70	0.04 (-0.05, 0.13)	0.71	-0.07 (-0.15, 0.02)	0.71	0.04 (-0.03, 0.12)	0.63	-0.06 (-0.13, 0.01)	0.70
N+WSH	0.05 (-0.02, 0.13)	0.51	NA	NA	-0.01 (-0.1, 0.07)	0.98	NA	NA	-0.04 (-0.13, 0.06)	0.66	NA	NA	-0.02 (-0.08, 0.04)	0.67	NA	NA
C. difficile																
WSH	0.01 (-0.04, 0.05)	0.86	0.02 (-0.02, 0.07)	0.75	-0.01 (-0.05, 0.04)	0.86	0.02 (-0.02, 0.07)	0.66	0.01 (-0.04, 0.05)	0.88	0.02 (-0.03, 0.07)	0.64	0 (-0.04, 0.05)	0.85	0.02 (-0.02, 0.07)	0.71
Nutrition	0.01 (-0.03, 0.06)	0.82	0.02 (-0.03, 0.06)	0.76	0.01 (-0.03, 0.06)	0.76	0.02 (-0.03, 0.06)	0.78	0.01 (-0.04, 0.06)	0.76	0.02 (-0.02, 0.07)	0.71	0.01 (-0.04, 0.06)	0.75	0.02 (-0.03, 0.06)	0.81
N+WSH	0.03 (-0.02, 0.08)	0.52	NA	NA	0.03 (-0.02, 0.09)	0.59	NA	NA	0.03 (-0.02, 0.08)	0.66	NA	NA	0.02 (-0.02, 0.07)	0.64	NA	NA
Plesiomonas																
WSH	0.01 (-0.05, 0.07)	0.86	0.04 (-0.02, 0.1)	0.63	-0.02 (-0.08, 0.03)	0.59	0.04 (-0.02, 0.1)	0.61	-0.02 (-0.08, 0.04)	0.61	0.05 (-0.01, 0.11)	0.37	-0.01 (-0.07, 0.04)	0.80	0.05 (-0.01, 0.11)	0.60
Nutrition	0.02 (-0.05, 0.08)	0.82	0.03 (-0.04, 0.1)	0.76	0 (-0.06, 0.06)	0.91	0.02 (-0.05, 0.09)	0.78	0.01 (-0.05, 0.08)	0.76	0.03 (-0.04, 0.1)	0.71	0.01 (-0.05, 0.08)	0.76	0.02 (-0.05, 0.09)	0.81
N+WSH	0.05 (-0.01, 0.11)	0.50	NA	NA	0.03 (-0.04, 0.09)	0.86	NA	NA	0.03 (-0.03, 0.1)	0.66	NA	NA	0.02 (-0.03, 0.08)	0.67	NA	NA
Shigella spp./EIEC																
WSH	0.01 (-0.05, 0.06)	0.86	0.02 (-0.04, 0.08)	0.76	-0.02 (-0.08, 0.03)	0.59	0.02 (-0.03, 0.08)	0.66	-0.04 (-0.1, 0.02)	0.47	0.03 (-0.03, 0.09)	0.64	-0.05 (-0.11, 0.01)	0.48	0.02 (-0.04, 0.08)	0.84
Nutrition	0.04 (-0.02, 0.11)	0.41	-0.02 (-0.08, 0.05)	0.76	0.03 (-0.04, 0.1)	0.66	-0.02 (-0.08, 0.04)	0.78	0 (-0.06, 0.06)	0.92	0 (-0.06, 0.06)	1.00	0.02 (-0.04, 0.08)	0.75	-0.02 (-0.09, 0.04)	0.81
N+WSH	0.03 (-0.03, 0.09)	0.53	NA	NA	0 (-0.05, 0.06)	1.00	NA	NA	-0.01 (-0.07, 0.04)	0.78	NA	NA	-0.02 (-0.07, 0.04)	0.73	NA	NA
**Adenovirus 40/41**																
WSH	-0.05 (-0.1, 0.01)	0.25	0.01 (-0.03, 0.05)	0.76	-0.09 (-0.15, -0.02)	0.05	0.01 (-0.03, 0.06)	0.74	-0.07 (-0.13, 0)	0.18	0.01 (-0.03, 0.05)	0.79	**-0.08 (-0.12, -0.04)**	**0.01**	0.03 (-0.03, 0.09)	0.71
Nutrition	-0.02 (-0.08, 0.04)	0.82	-0.01 (-0.06, 0.04)	0.76	-0.04 (-0.11, 0.03)	0.66	-0.01 (-0.06, 0.04)	0.79	-0.03 (-0.1, 0.05)	0.76	-0.02 (-0.07, 0.03)	0.76	-0.05 (-0.1, 0)	0.36	0 (-0.06, 0.05)	0.99
N+WSH	-0.03 (-0.09, 0.02)	0.52	NA	NA	-0.06 (-0.14, 0.01)	0.54	NA	NA	-0.05 (-0.12, 0.02)	0.64	NA	NA	-0.06 (-0.1, -0.01)	0.10	NA	NA
**Norovirus**																
WSH	**-0.11 (-0.17, -0.06)**	**<0.01**	0.06 (0.01, 0.12)	0.22	**-0.12 (-0.19, -0.06)**	**<0.01**	0.06 (0.01, 0.12)	0.23	**-0.1 (-0.16, -0.05)**	**0.01**	0.05 (-0.01, 0.11)	0.37	**-0.11 (-0.17, -0.05)**	**0.01**	0.06 (0.01, 0.12)	0.46
Nutrition	-0.05 (-0.12, 0.02)	0.41	0 (-0.06, 0.07)	0.95	-0.04 (-0.12, 0.04)	0.66	0.01 (-0.06, 0.07)	0.89	-0.03 (-0.11, 0.05)	0.76	0 (-0.07, 0.07)	1.00	-0.03 (-0.09, 0.03)	0.68	0 (-0.06, 0.06)	0.99
N+WSH	-0.05 (-0.12, 0.02)	0.50	NA	NA	-0.04 (-0.12, 0.04)	0.75	NA	NA	-0.06 (-0.13, 0.01)	0.64	NA	NA	-0.05 (-0.12, 0.02)	0.36	NA	NA
**Sapovirus**																

(continued)

Pathogen	Unadjusted Model				Age & Season Adjusted Model				Fully Adjusted Model w/ non-diarrheal samples[a]				IPCW Adjusted Model[b]			
	Intervention v Control		N+WSH v Intervention		Intervention v Control		N+WSH v Intervention		Intervention v Control		N+WSH v Intervention		Intervention v Control		N+WSH v Intervention	
Arm	PD (95% CI)	p[c]	PD (95% CI)	p[c]	PD (95% CI)	p[c]	PD (95% CI)	p[c]	PD (95% CI)	p[c]	PD (95% CI)	p[c]	PD (95% CI)	p[c]	PD (95% CI)	p[c]
WSH	**-0.11 (-0.17, -0.05)**	**<0.01**	0.03 (-0.02, 0.08)	0.63	**-0.1 (-0.17, -0.03)**	**0.02**	0.03 (-0.02, 0.08)	0.61	-0.08 (-0.15, -0.01)	0.15	0.05 (0, 0.1)	0.37	-0.04 (-0.12, 0.04)	0.69	-0.01 (-0.08, 0.07)	0.86
Nutrition	-0.09 (-0.15, -0.03)	0.06	0.02 (-0.04, 0.07)	0.76	-0.08 (-0.15, -0.02)	0.15	0.02 (-0.03, 0.07)	0.78	-0.07 (-0.13, 0)	0.57	0.03 (-0.03, 0.08)	0.71	**-0.08 (-0.13, -0.03)**	**0.03**	0.02 (-0.02, 0.07)	0.81
N+WSH	-0.08 (-0.14, -0.01)	0.14	NA	NA	-0.06 (-0.12, 0.01)	0.54	NA	NA	-0.03 (-0.1, 0.04)	0.66	NA	NA	-0.06 (-0.11, 0)	0.22	NA	NA
Cryptosporidium																
WSH	**0.07 (0.02, 0.11)**	**0.03**	-0.01 (-0.06, 0.05)	0.84	0.02 (-0.03, 0.07)	0.59	-0.01 (-0.06, 0.05)	0.84	0.03 (-0.02, 0.09)	0.47	-0.02 (-0.07, 0.03)	0.64	0.01 (-0.04, 0.06)	0.80	-0.01 (-0.06, 0.05)	0.86
Nutrition	0.04 (0, 0.09)	0.24	0.02 (-0.04, 0.07)	0.76	0.02 (-0.03, 0.07)	0.66	0.01 (-0.04, 0.07)	0.79	0.03 (-0.02, 0.09)	0.64	0 (-0.05, 0.05)	1.00	0.02 (-0.02, 0.06)	0.68	0.01 (-0.04, 0.06)	0.82
N+WSH	0.06 (0.01, 0.11)	0.14	NA	NA	0.04 (-0.02, 0.09)	0.59	NA	NA	0.04 (-0.01, 0.1)	0.64	NA	NA	0.05 (0, 0.1)	0.22	NA	NA
E. bieneusi																
WSH	-0.01 (-0.06, 0.04)	0.86	-0.01 (-0.05, 0.04)	0.81	-0.02 (-0.07, 0.04)	0.72	-0.01 (-0.05, 0.04)	0.80	-0.02 (-0.08, 0.03)	0.61	0 (-0.05, 0.05)	0.94	-0.02 (-0.07, 0.03)	0.69	-0.01 (-0.05, 0.04)	0.86
Nutrition	0.01 (-0.04, 0.06)	0.90	-0.03 (-0.07, 0.02)	0.76	-0.01 (-0.06, 0.04)	0.80	-0.03 (-0.08, 0.02)	0.70	-0.03 (-0.08, 0.02)	0.64	-0.02 (-0.07, 0.02)	0.71	-0.01 (-0.06, 0.04)	0.76	-0.03 (-0.07, 0.01)	0.80
N+WSH	-0.02 (-0.06, 0.03)	0.60	NA	NA	-0.02 (-0.07, 0.03)	0.86	NA	NA	-0.03 (-0.08, 0.02)	0.64	NA	NA	-0.03 (-0.08, 0.01)	0.36	NA	NA
Giardia																
WSH	0.01 (-0.05, 0.06)	0.86	-0.02 (-0.07, 0.03)	0.76	0.02 (-0.03, 0.08)	0.59	-0.02 (-0.07, 0.03)	0.66	-0.02 (-0.08, 0.04)	0.61	-0.01 (-0.06, 0.04)	0.73	-0.01 (-0.06, 0.05)	0.82	-0.02 (-0.07, 0.03)	0.71
Nutrition	0 (-0.06, 0.06)	1.00	-0.01 (-0.06, 0.04)	0.76	0.03 (-0.04, 0.09)	0.66	-0.01 (-0.06, 0.05)	0.89	-0.04 (-0.1, 0.02)	0.64	0.01 (-0.04, 0.06)	0.94	-0.02 (-0.07, 0.04)	0.75	-0.01 (-0.06, 0.04)	0.82
N+WSH	-0.01 (-0.06, 0.04)	0.79	NA	NA	0.01 (-0.05, 0.06)	0.98	NA	NA	-0.03 (-0.09, 0.02)	0.66	NA	NA	-0.02 (-0.07, 0.03)	0.67	NA	NA

IPCW, inverse probability of censoring weighting; NA, not applicable; PD, Prevalence difference; WSH, water, sanitation, and handwashing; N+WSH, nutrition plus water, sanitation, and handwashing

[a] Non-diarrheal samples include those from children with no reported diarrheal symptoms in the previous seven days.

[b] Adjusted for pre-specified covariates with a likelihood ratio test $p < 0.1$ in bivariate analysis with the outcome: household food insecurity; child age, sex, and birth order; season of sample collection; time until sample placed on cold chain; mother's age, height, and education level; number of children < 18 years in the household; number of individuals living in the compound; distance in minutes to the primary water source; household floor & wall materials; household assets.

[c] p values shown are adjusted for false discovery rate using the Benjamini-Hochberg procedure.

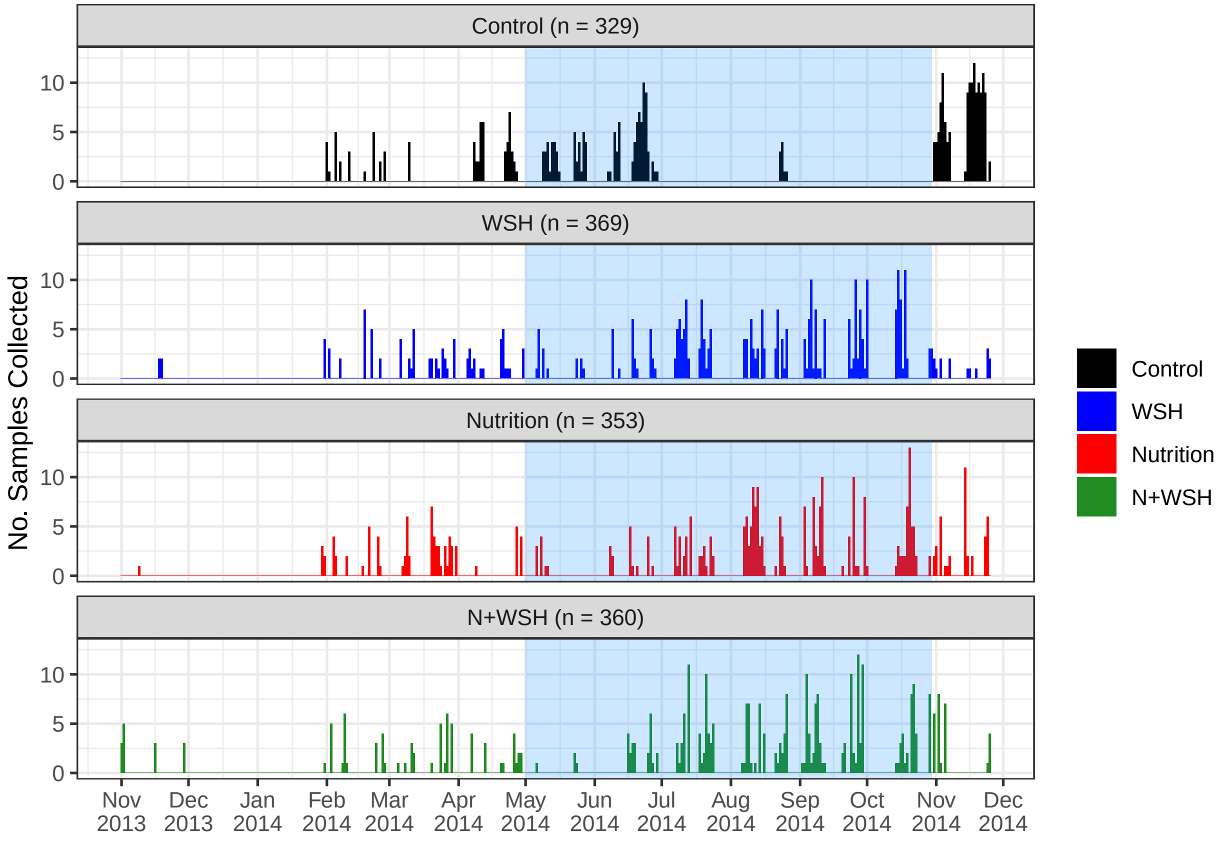

Figure S1: **Timing of fecal sample collection for the WASH Benefits Bangladesh Environmental Enteropathy cohort (n = 1411).** Sample collection was disrupted in all arms due to political instability surrounding the Jan 2014 presidential election, and primarily in the control arm during subsequent unrest in July-Oct 2014 amid calls for a re-vote due to corruption. Blue overlay indicates monsoon season.

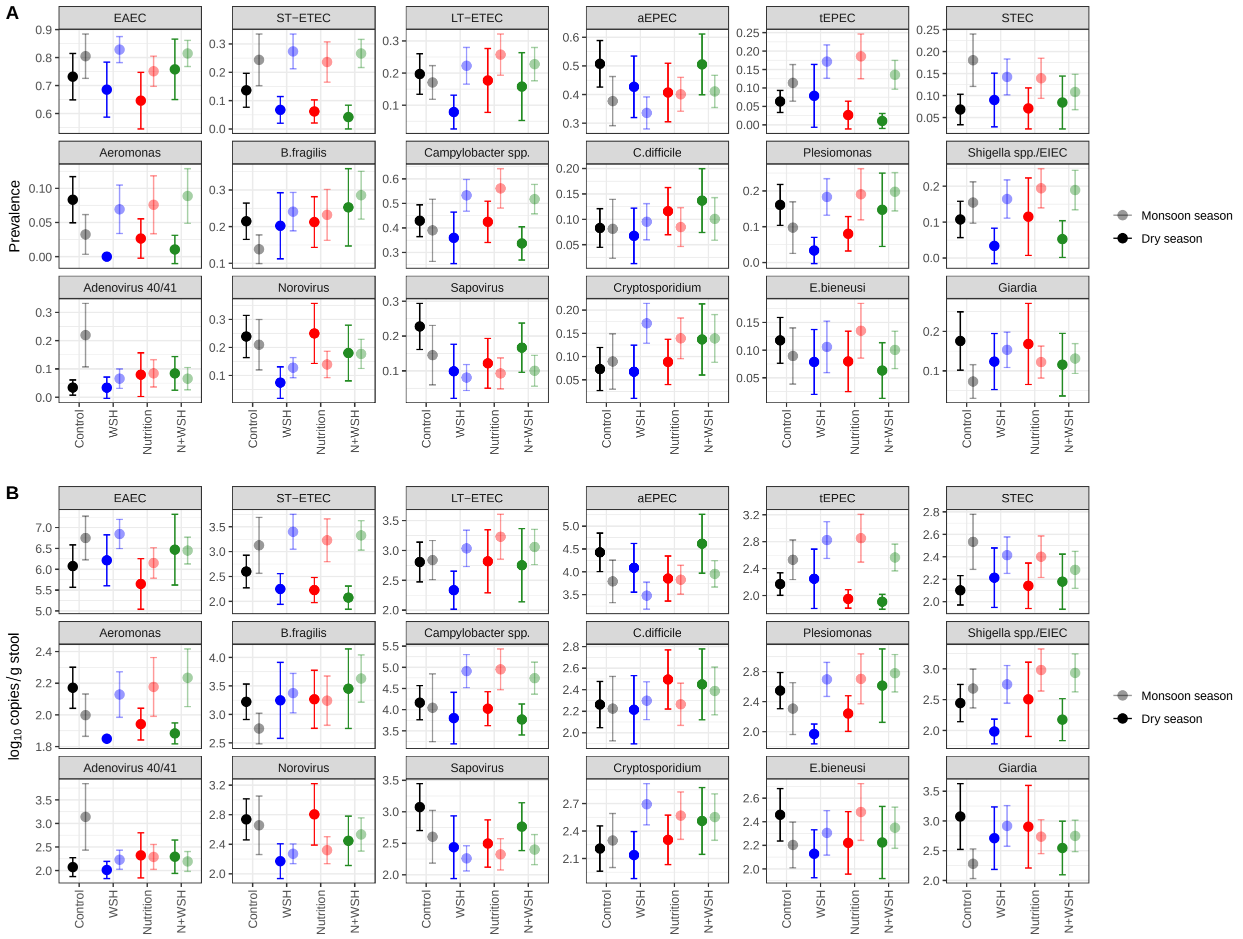

Figure S2: **Some enteric pathogens in 14 month old children influenced by monsoon season.** Difference in individual pathogen A) prevalence and B) $\log_{10}$ quantity between seasons. Bold circles show prevalence during dry season and muted circles show prevalence during monsoon season.

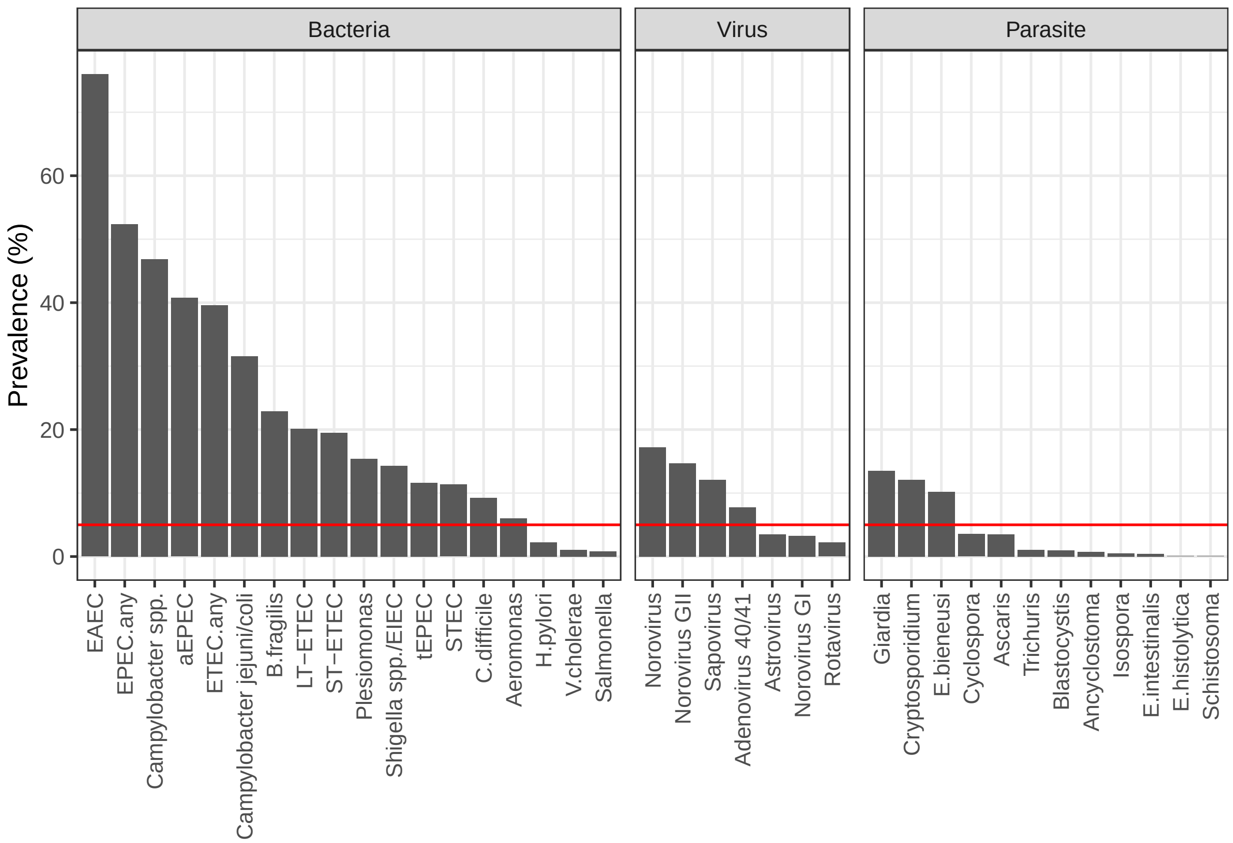

Figure S3: **Prevalence of enteric pathogens in 14 month old children from rural Bangladesh** Prevalence of pathogens across all study arms (n=1411). Red line indicates the 5% prevalence cutoff used to determine if pathogen would be included in individual pathogen analyses. Plot also shows aggregated values for EPEC (EPEC.any = aEPEC + tEPEC), ETEC (ETEC.any = ST-ETEC + LT-ETEC), *Campylobacter* spp. (also showing prevalence of *C. jejuni/coli*), and Norovirus (Norovirus = Norovirus GI + Norovirus GII)

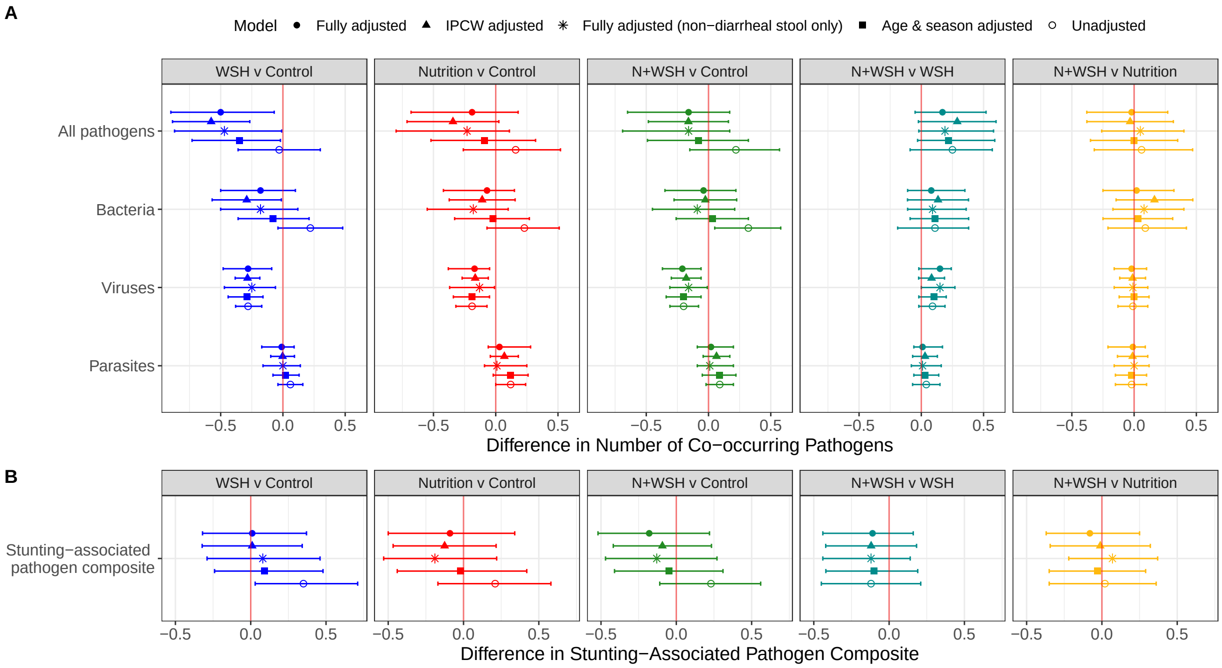

Figure S4: **Difference in composite pathogen metrics between intervention arms in children aged 14 months old.** Difference in A) number of co-occurring enteric pathogens, reported as all pathogens and by type: bacteria, viruses, or parasites; and B) the stunting-associated composite score. Data points represent the point estimate and error bars indicate the 95% confidence intervals. Shapes indicate model adjustment scenarios: Fully adjusted for pre-specified covariates; Inverse probability of censoring weighting (IPCW) adjusted for potential bias due to informative censoring and including adjustment for pre-specified covariates; Fully adjusted for pre-specified covariates but only including stool samples from children without reported diarrhea in the past 7 days; Adjusted for child age and season of sample collection only; Unadjusted.

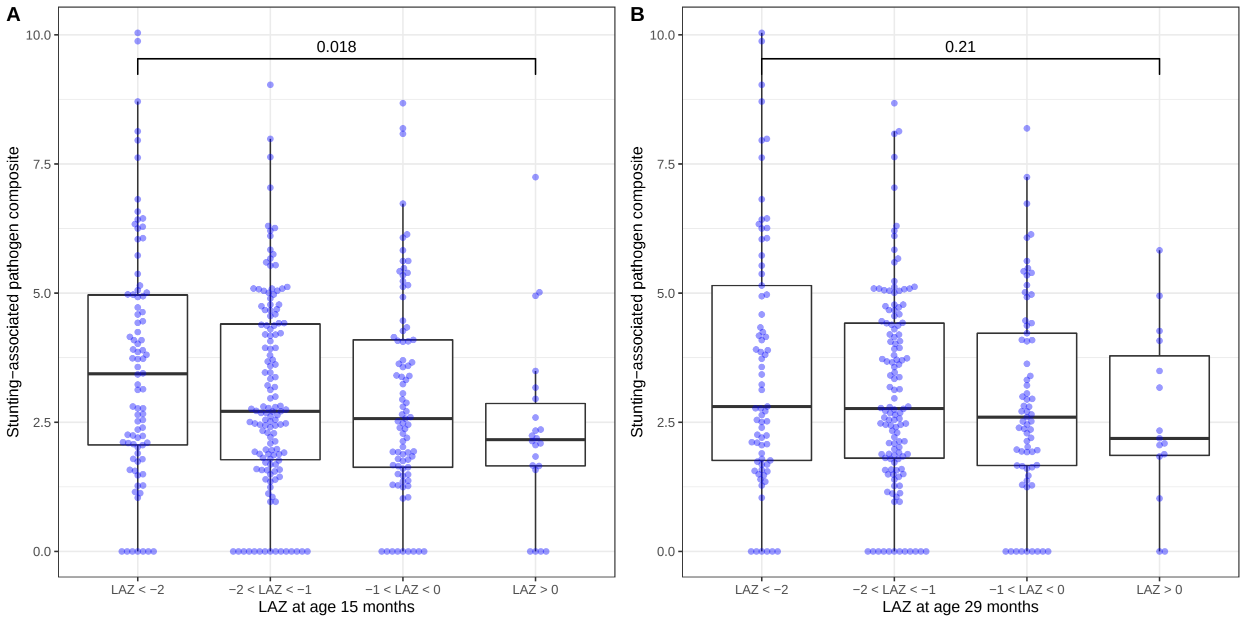

Figure S5: **Stunting-associated pathogen composite score is associated with concurrent, but not future, child length-for-age Z score (LAZ).** Each point represents a child and plots show the quantitative composite of four stunting-associated enteric pathogens (EAEC, *Shigella* spp., *Campylobacter* spp, and *Giardia*) measured at age 15 months compared to A) LAZ at 15 months and B) LAZ at 29 months. P-values calculated with the nonparametric Wilcoxon rank-sum test. LAZ was calculated from triplicate measurements of recumbent length (to 0.1 cm) taken during a household visit by trained anthropometrists according to standard protocols described in the main trial protocol.

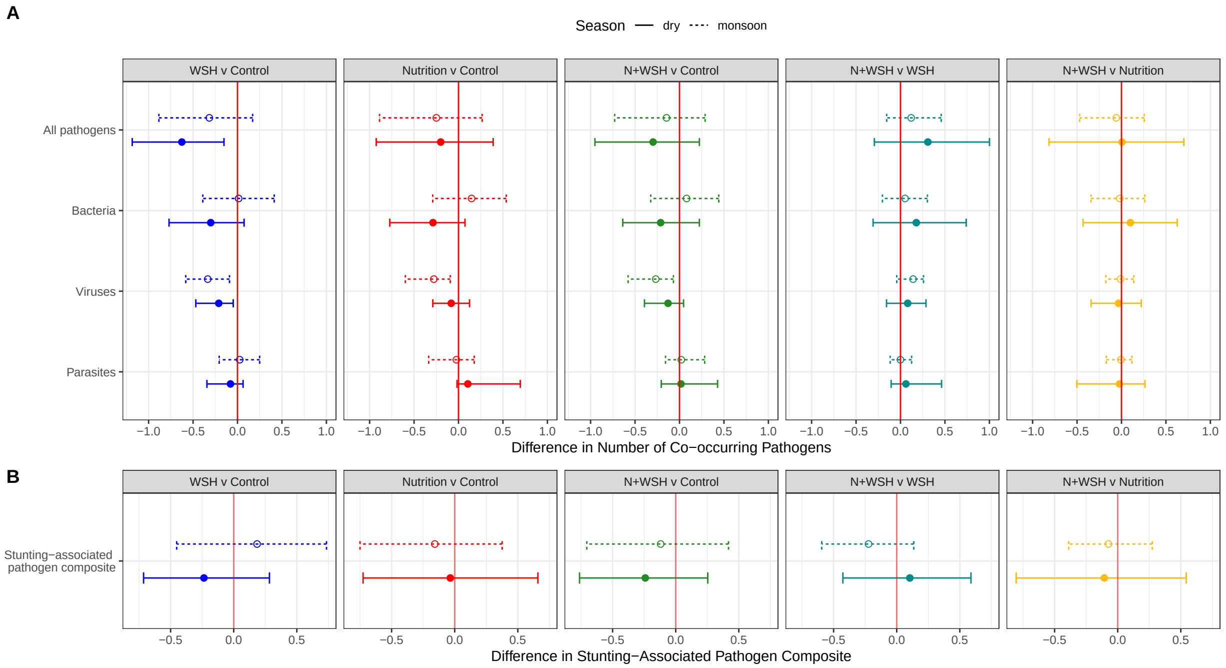

Figure S6: **Difference in composite pathogen metrics between intervention arms by season.** Difference in A) number of co-occurring enteric pathogens, reported as all pathogens and by type: bacteria, viruses, or parasites; and B) the stunting-associated composite score. Point estimates and 95% confidence intervals determined with a parametric g-formula using a generalized linear model including an interaction term for season of sample collection and adjusting for covariates associated with each pathogen outcome (likelihood ratio test $p < 0.1$ in bivariate analysis): household food insecurity, child age, child sex, child birth order, time between defecation and sample placed on cold chain, mother's age, mothers height, mothers education level, number of children <18 years in the household, number of individuals living in the compound, distance in minutes to the primary water source, household floor & wall materials, household assets.

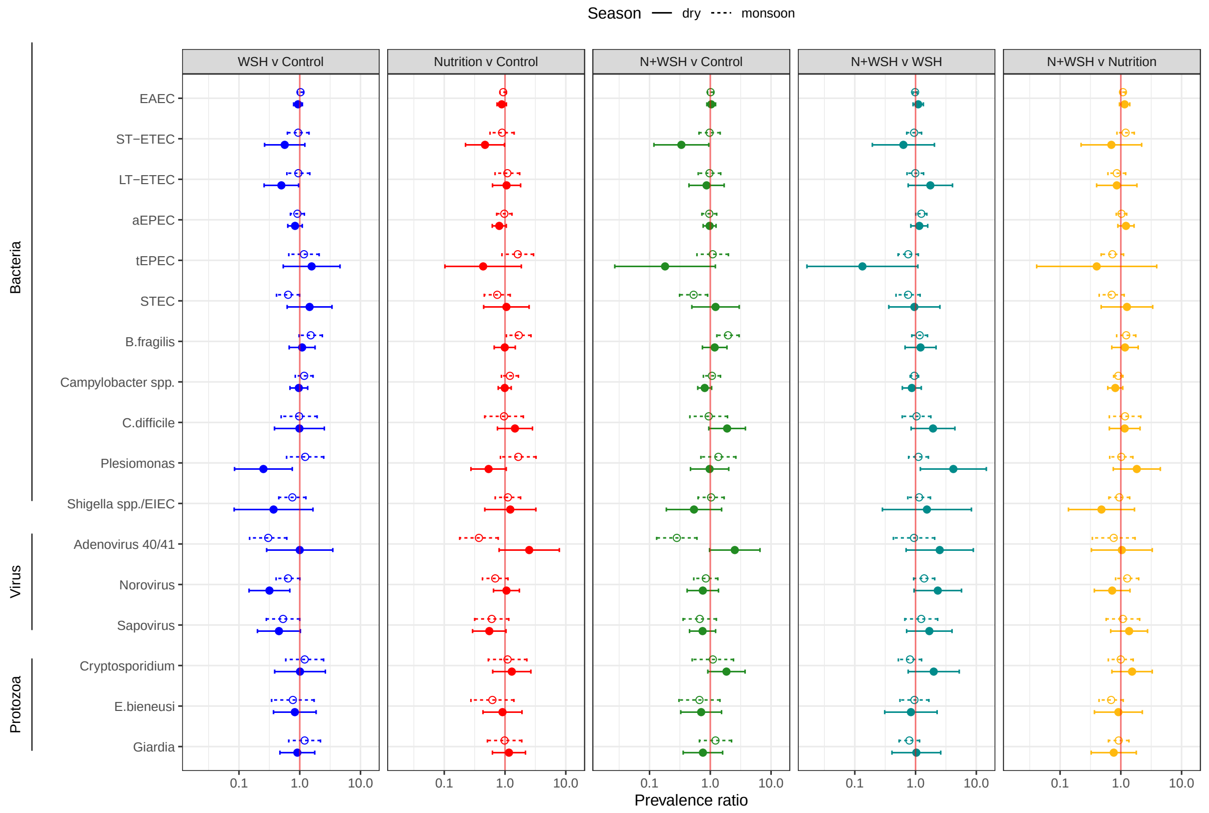

Figure S7: **Difference in pathogen prevalence by season.** Point estimates and 95% confidence intervals determined with a generalized linear model including an interaction term for season of sample collection and adjusting for covariates associated with each pathogen outcome (likelihood ratio test $p < 0.1$ in bivariate analysis): household food insecurity, child age, child sex, child birth order, time between defecation and sample placed on cold chain, mother's age, mothers height, mothers education level, number of children <18 years in the household, number of individuals living in the compound, distance in minutes to the primary water source, household floor & wall materials, household assets. *Aeromonas* is not reported as the model failed due to sparsity. No pathogen had significantly lower prevalence in children receiving any intervention compared to control children for either season after adjusting for false discovery rate ($p < 0.01$).

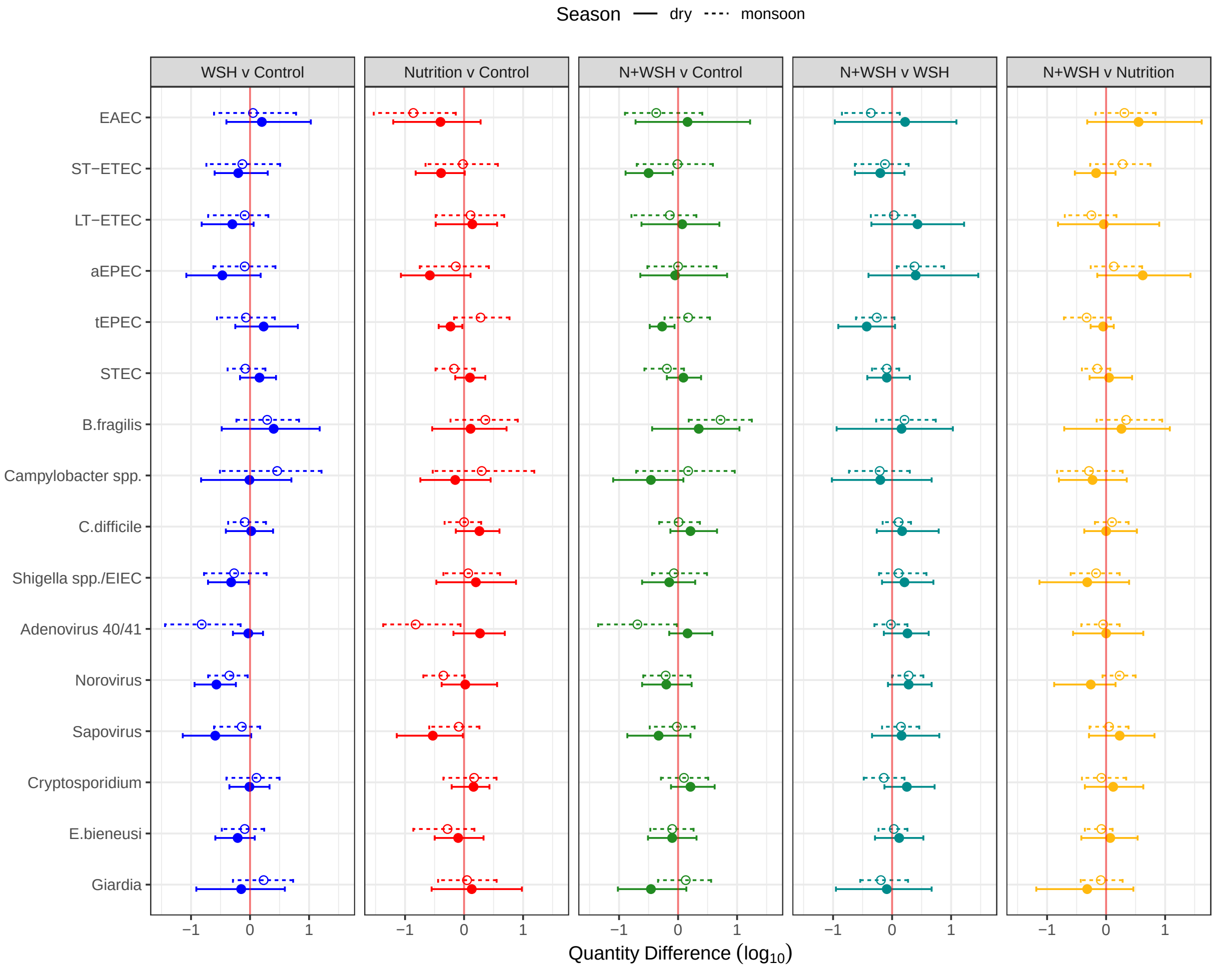

Figure S8: **Difference in pathogen quantity ($\log_{10}$ copies/ gram of stool) by season.** Point estimates and 95% confidence intervals determined with a parametric g-formula including both logistic and log-linear regression steps with generalized linear models including an interaction term for season of sample collection and adjusting for covariates associated with each pathogen outcome (likelihood ratio test $p < 0.1$ in bivariate analysis): household food insecurity, child age, child sex, child birth order, time between defecation and sample placed on cold chain, mother's age, mothers height, mothers education level, number of children <18 years in the household, number of individuals living in the compound, distance in minutes to the primary water source, household floor & wall materials, household assets. *Aeromonas* and *Plesiomonas* are not reported as the model failed in a large number of bootstraps due to sparsity. After adjusting for false discovery rate the only pathogen that had significantly lower ($p < 0.01$) quantity was tEPEC in the dry season for children in the N+WSH arm compared to control children.

References

[1] Jie Liu, Jean Gratz, Caroline Amour, Gibson Kibiki, Stephen Becker, Lalitha Janaki, Jaco J Verweij, Mami Taniuchi, Shihab U Sobuz, Rashidul Haque, Doris M Haverstick, and Eric R Houpt. A laboratory-developed TaqMan Array Card for simultaneous detection of 19 enteropathogens. *Journal of Clinical Microbiology*, 51(2):472–80, mar 2013.

[2] Jie Liu, James A Platts-Mills, Jane Juma, Furqan Kabir, Joseph Nkeze, Catherine Okoi, et al. Use of quantitative molecular diagnostic methods to identify causes of diarrhoea in children: a reanalysis of the GEMS case-control study. *The Lancet*, 388(10051):1291–1301, 2016.

[3] Jie Liu, Jean Gratz, Caroline Amour, Rosemary Nshama, Thomas Walongo, Athanasia Maro, Esto Mduma, James Platts-Mills, Nadia Boisen, James Nataro, Doris M. Haverstick, Furqan Kabir, Paphavee Lertsethtakarn, Sasikorn Silapong, Pimmada Jeamwattanalert, Ladaporn Bodhidatta, Carl Mason, Sharmin Begum, Rashidul Haque, Ira Praharaj, Gagandeep Kang, and Eric R. Houpt. Optimization of quantitative PCR methods for enteropathogen detection. *PLoS ONE*, 11(6):1–11, 2016.
